# Supplementary material for: Protein arginine deiminase 4 antagonizes methylglyoxal-induced histone glycation
Source: Nat Commun. 2020 Jun 26;11:3241. doi: 10.1038/s41467-020-17066-y (PMC7319962; doi:10.1038/s41467-020-17066-y)
Supplement: Supplementary file 1 — Supplementary Information [file 41467_2020_17066_MOESM1_ESM.pdf]

Protein Arginine Deiminase 4 Antagonizes Methylglyoxal-induced Histone Glycation

Zheng et al.

Supplementary figures

Supplementary Figure 1. SDS-PAGE analysis of PAD4 purification. The lanes from left to right are protein marker (M), concentrated PAD4 stock used for the *in vitro* assays (1), PAD4 before concentration (2), cleaved PAD4 before reverse GST column purification (3) and purified PAD4 before tag cleavage (4), respectively.

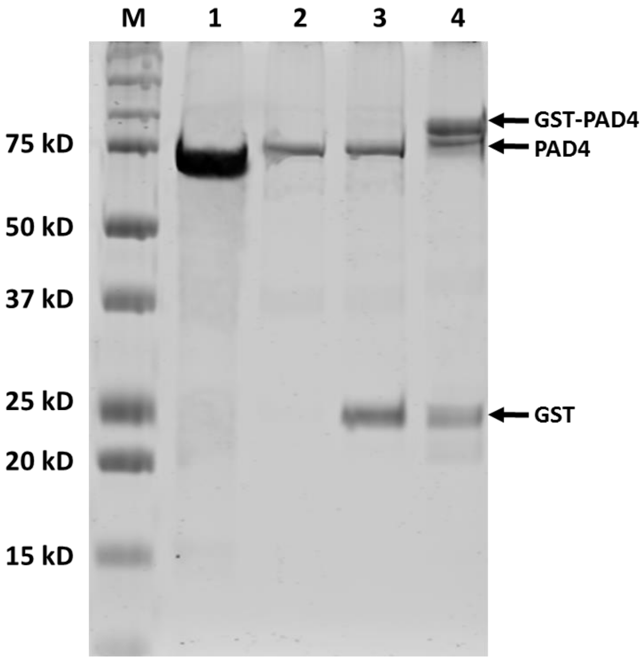

Supplementary Figure 2. Differences of PAD4 enzymatic activity against distinct substrates (free histone H3, nucleosome core particles and nucleosomal arrays). Free histone H3, NCPs and nucleosomal arrays were treated with PAD4 at 37 °C for 2 h, and then separated by SDS-PAGE followed by western blot analysis.

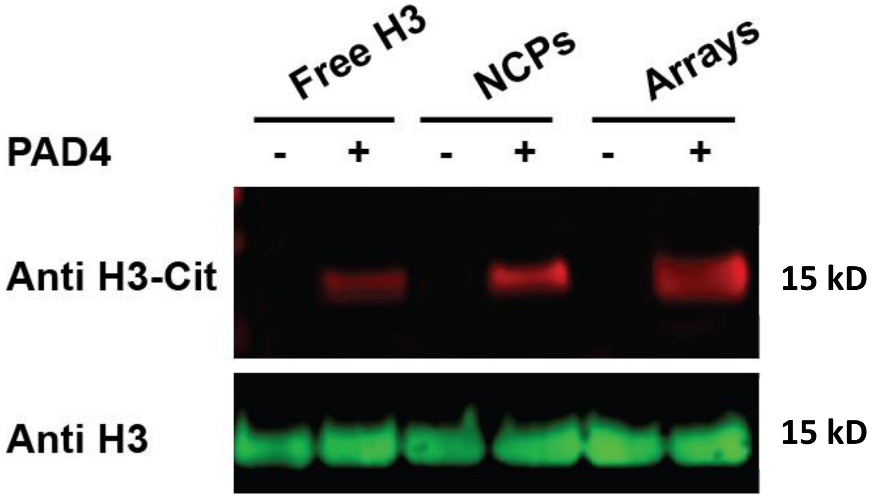

Supplementary Figure 3. Ca^{2+}-dependent assays of PAD4 citrullination. The NCP citrullination reactions were performed in the presence or absence of Ca^{2+} under 37 °C for 2 h, and then separated by SDS-PAGE followed by western blot analysis.

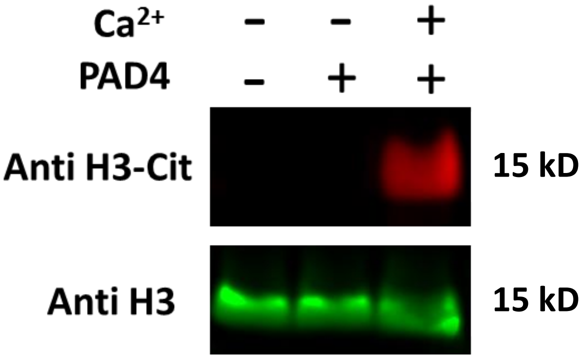

Supplementary Figure 4. Dose-dependent NCP citrullination and glycation. NCPs were treated with gradient PAD4 under 37 °C for 2 h, followed by MGO treatment under 37 °C overnight, and then analyzed by native gel electrophoresis and western blot. The stoichiometry of enzyme and substrate is 0:20, 1:20, 2:20 and 4:20.

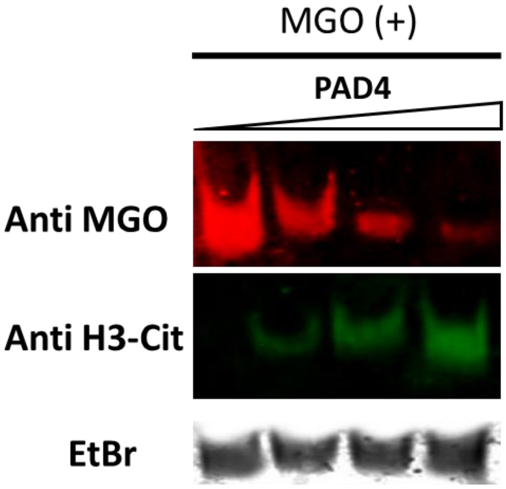

Supplementary Figure 5. LC-MS analysis of MGO-induced glycation and PAD4-mediated deglycation of H3 N-terminal peptide substrate. The amino acid sequence of the peptide is ARTKQTARKSTGGKAPRK(Bio)A (calculated M=2237.3). (a) Ultraviolet absorption (λ=214 nm) of modified and unmodified peptides: **1** unmodified peptide (i), **2** citrullinated peptide by PAD4 in H_2O (ii and iv) or $H_2{}^{18}O$ (v), and **3** glycated peptide by MGO (iii). (b) Mass spectrometric analysis of corresponding peaks in A: **1** in i, M=2237.8; **2** in ii, M=2238.0; **3** in iii, M=2310.4; **2** in iv, M=2238.2; **2** in v, M=2240.2.

a

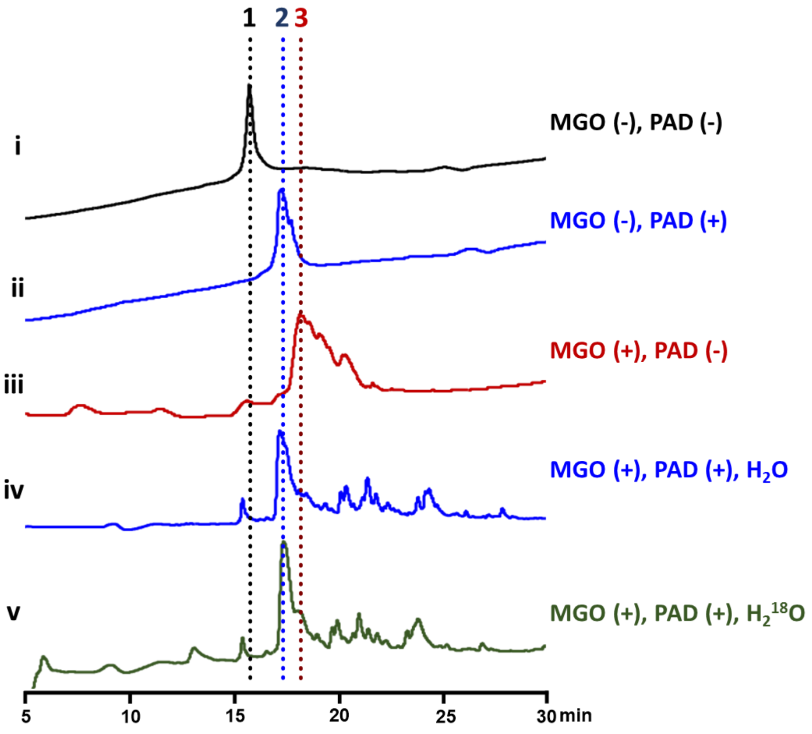

b

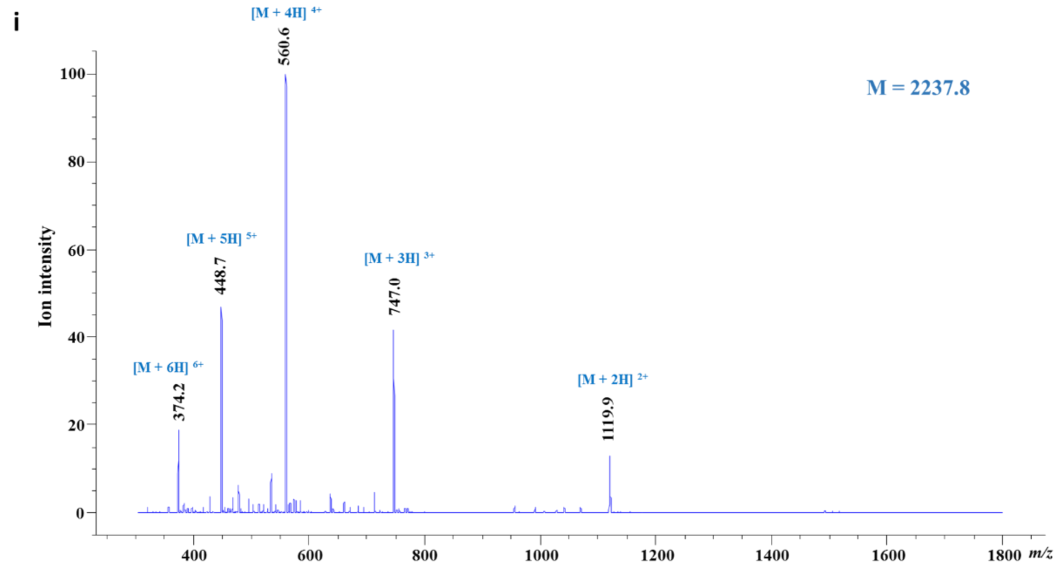

ii

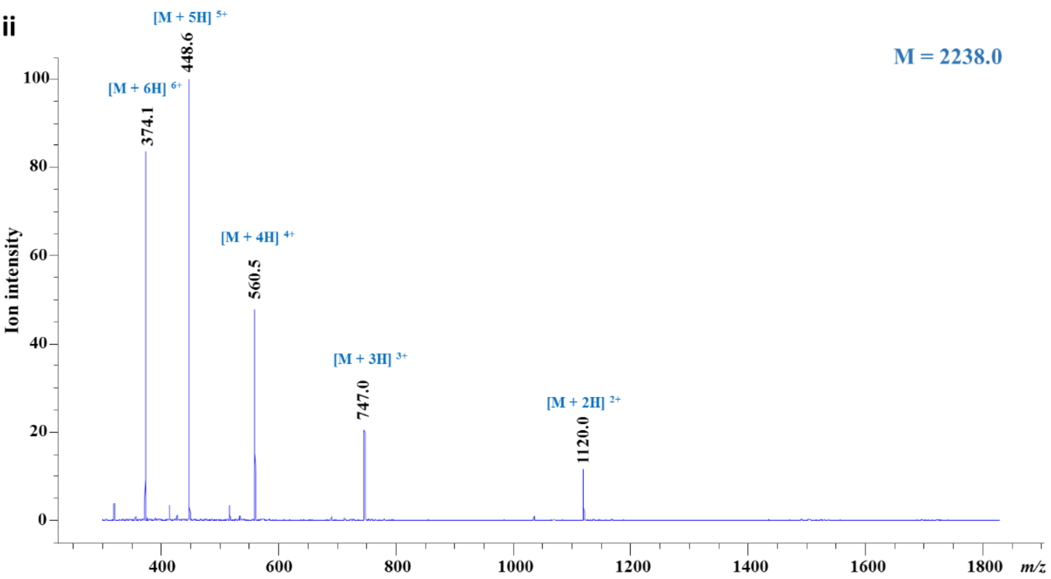
[M + 5H] 5+
448.6
[M + 6H] 6+
374.1
[M + 4H] 4+
560.5
[M + 3H] 3+
747.0
[M + 2H] 2+
1120.0
M = 2238.0
Ion intensity
m/z

iii

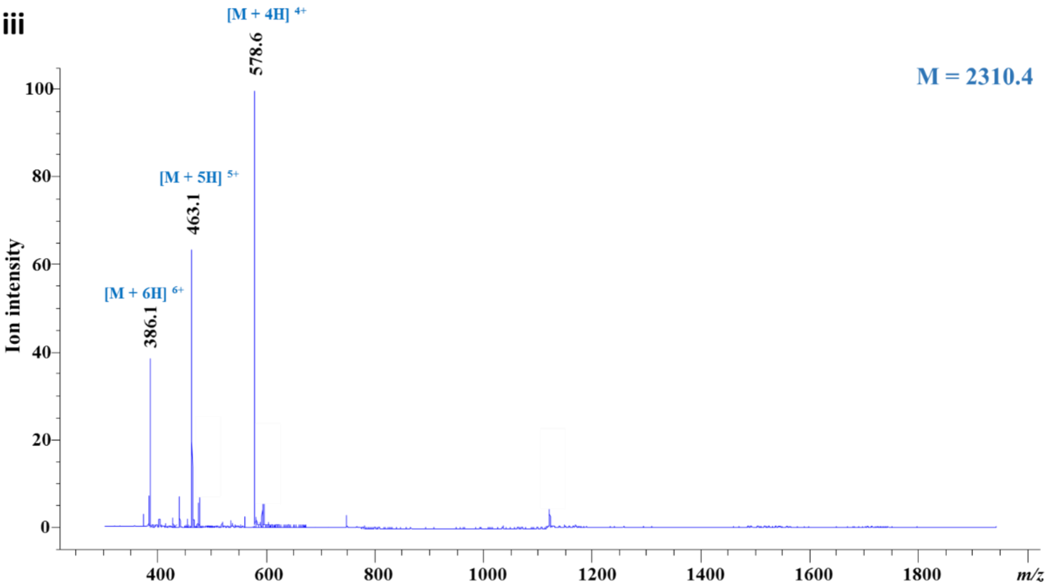
[M + 4H] 4+
578.6
[M + 5H] 5+
463.1
[M + 6H] 6+
386.1
M = 2310.4
Ion intensity
m/z

iv

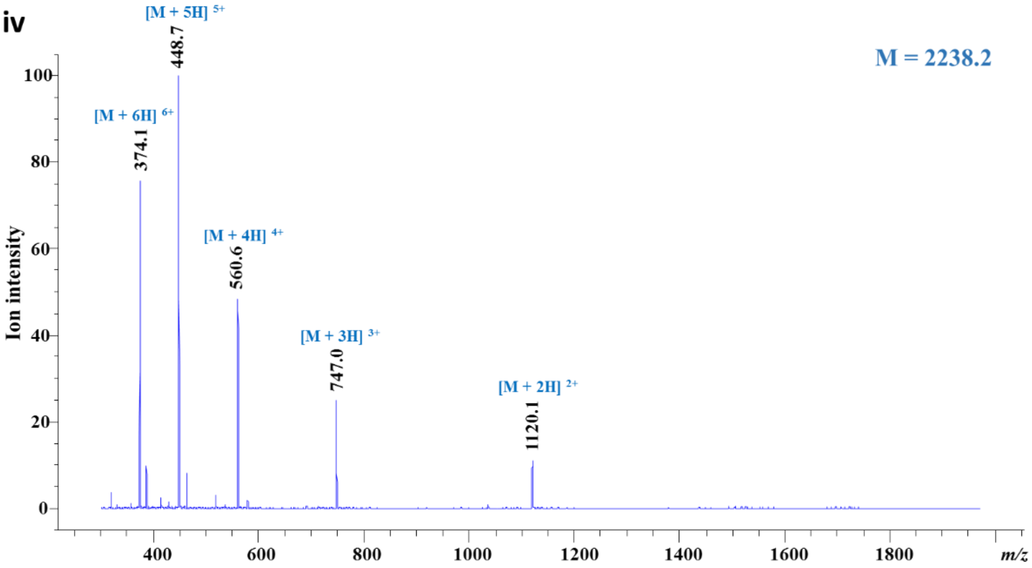
[M + 5H] 5+
448.7
M = 2238.2
[M + 6H] 6+
374.1
[M + 4H] 4+
560.6
[M + 3H] 3+
747.0
[M + 2H] 2+
1120.1
Ion intensity
m/z

v

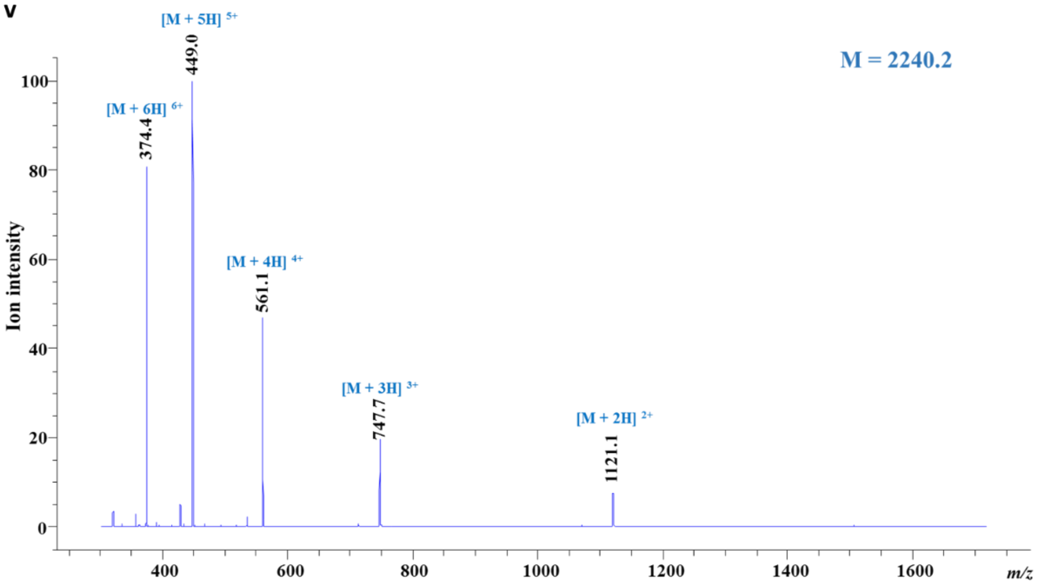
[M + 5H] 5+
449.0
M = 2240.2
[M + 6H] 6+
374.4
[M + 4H] 4+
561.1
[M + 3H] 3+
747.7
[M + 2H] 2+
1121.1
Ion intensity
m/z

Supplementary Figure 6. LC-MS analysis of MGO-induced glycation and PAD4-mediated deglycation on H4 N-terminal peptide substrate. H4-R3(1-6) (calculated Mass=827.4) and H4-Cit3(1-6) (calculated Mass=828.4) were synthesized by SPPS as described in the Methods section and were used as substrate and product standard, respectively. MS analysis of the corresponding peaks in Figure 2e: a) M=827.7 (i), b) M=828.5 (ii), c) M=899.4 (iii), d) M=830.4 (iv).

a

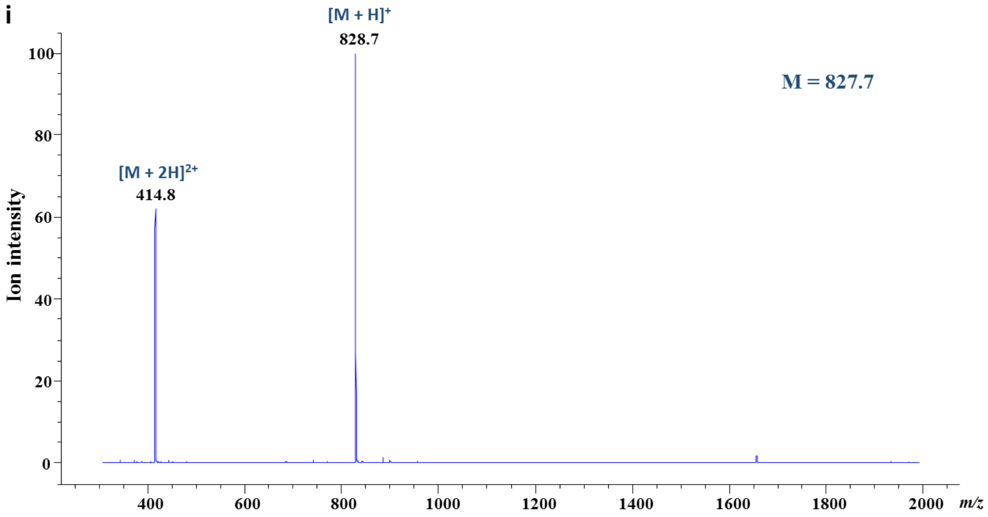

b

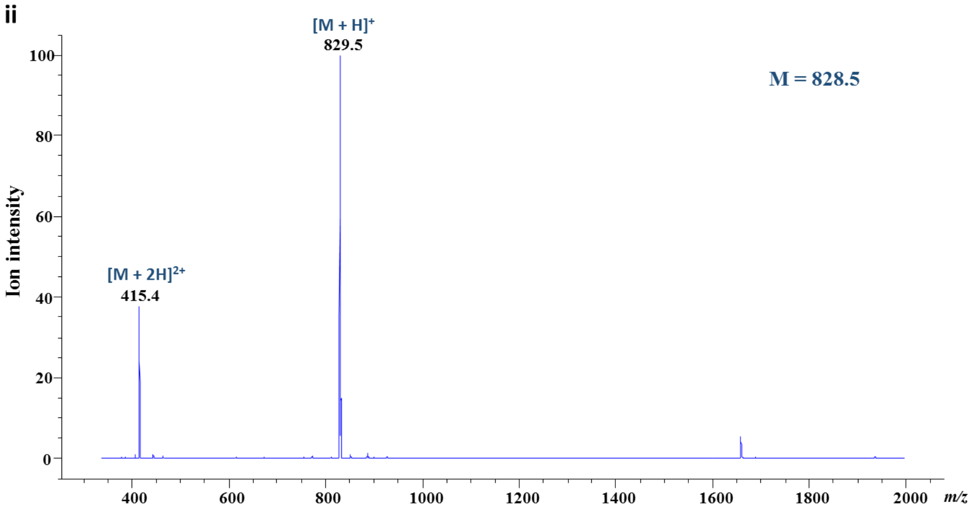

c

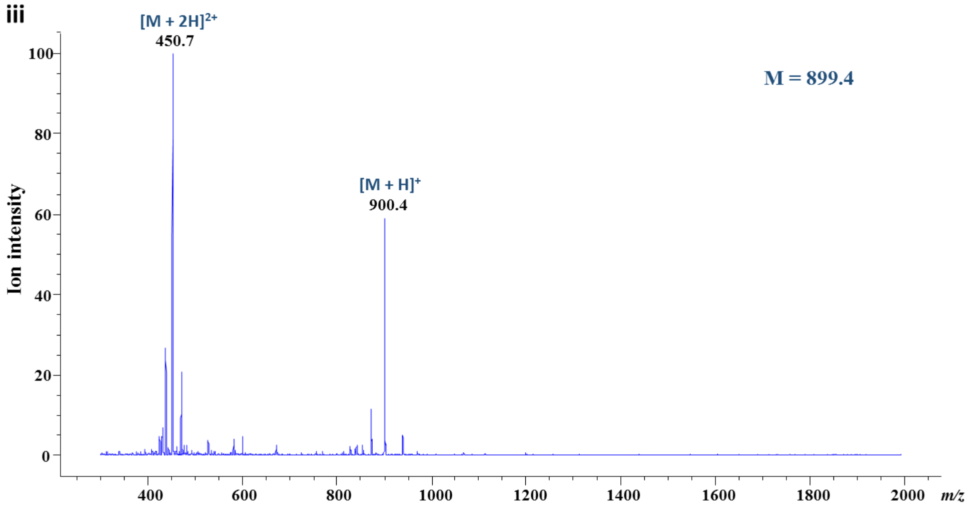
iii
[M + 2H]2+
450.7
[M + H]+
900.4
M = 899.4
Ion intensity
m/z
0
20
40
60
80
100
400
600
800
1000
1200
1400
1600
1800
2000

d

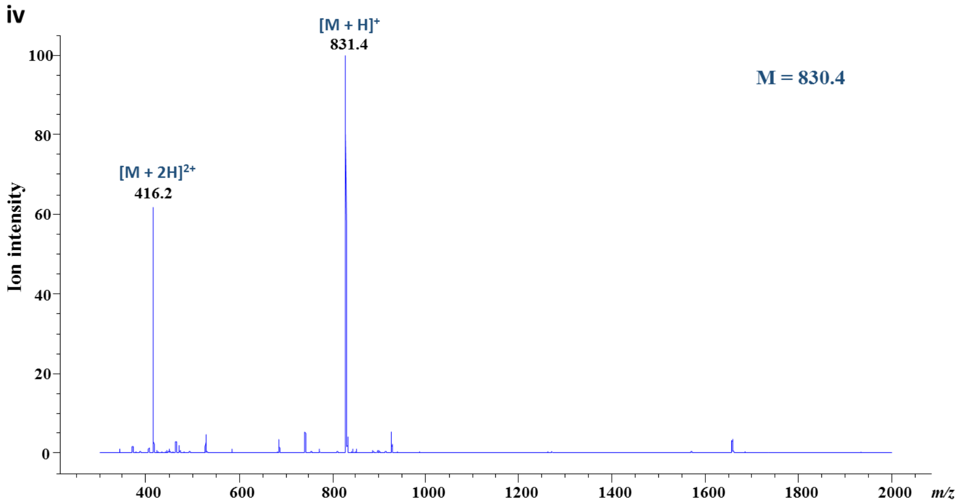
iv
[M + H]+
831.4
[M + 2H]2+
416.2
M = 830.4
Ion intensity
m/z
0
20
40
60
80
100
400
600
800
1000
1200
1400
1600
1800
2000

Supplementary Figure 7. Comparison of PAD4 and PRMT1 activities on MGO-glycated and full-length histone H4. Recombinant H4 was incubated with (or without) MGO at 37 °C for 2 h and treated with PAD4 or PRMT1, followed by SDS-PAGE and western blot analysis with the indicated antibodies.

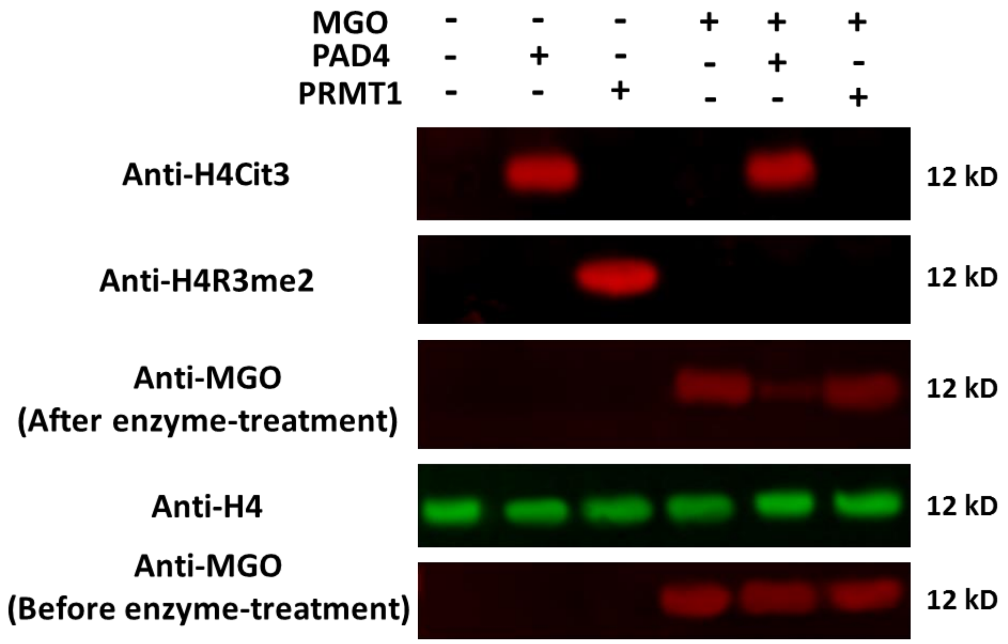

Supplementary Figure 8. Deglycase activities of DJ-1 and PAD4 on nucleosomal arrays. (a) The arrays were pre-treated with 5 mM MGO for short (S, 6 h) and long period (L, 6 h + 12 h) in the presence or absence of enzymes. The enzymes were co-incubated with arrays and MGO (Co), incubated with glycated arrays after short treatment of MGO (S) or incubated with glycated arrays after long treatment of MGO (L); (b) Combined treatment of WT or mutant enzymes (DJ1-C106A and PAD4-C645S) to glycated nucleosomal arrays. Error bars represent the standard deviation from three different experiments. Data are presented as mean values +/- SEM.

a

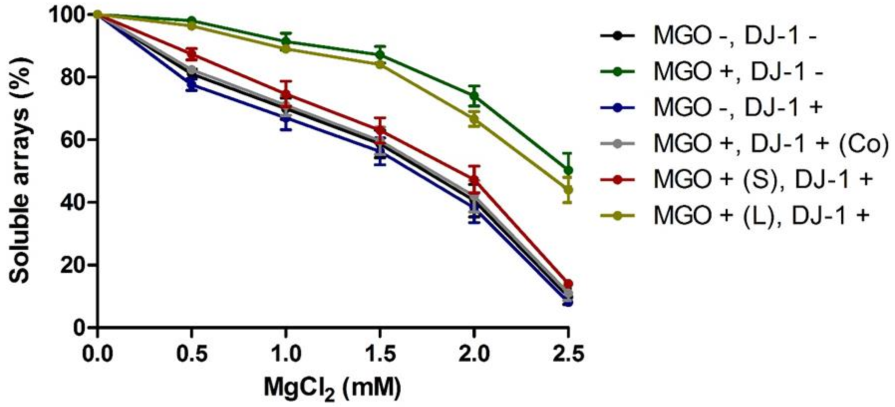

b

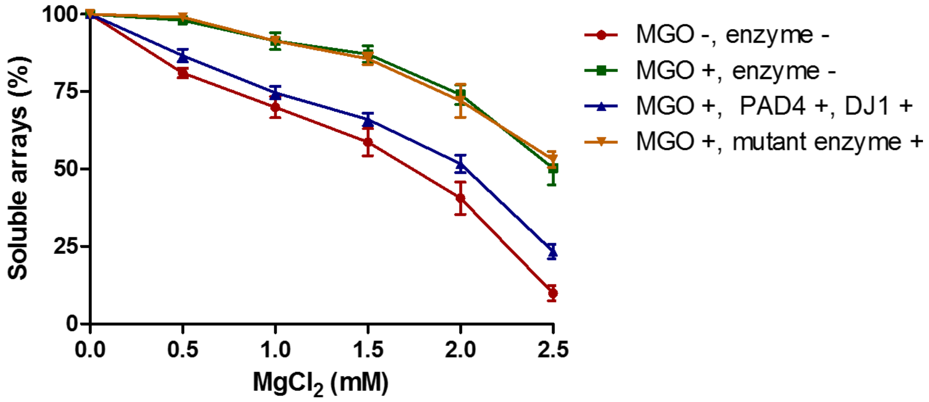

Supplementary Figure 9. SDS-PAGE western blot analysis of extracted histones from wild type (left) and PAD4-overexpressing (right) 293T cells. Histones were extracted using salt extraction protocol described in supplementary method section. The gel was stained by Coomassie Brilliant Blue (CBB), and the histones were also transferred to a PVDF membrane and blotted with pan anti-citrulline antibody.

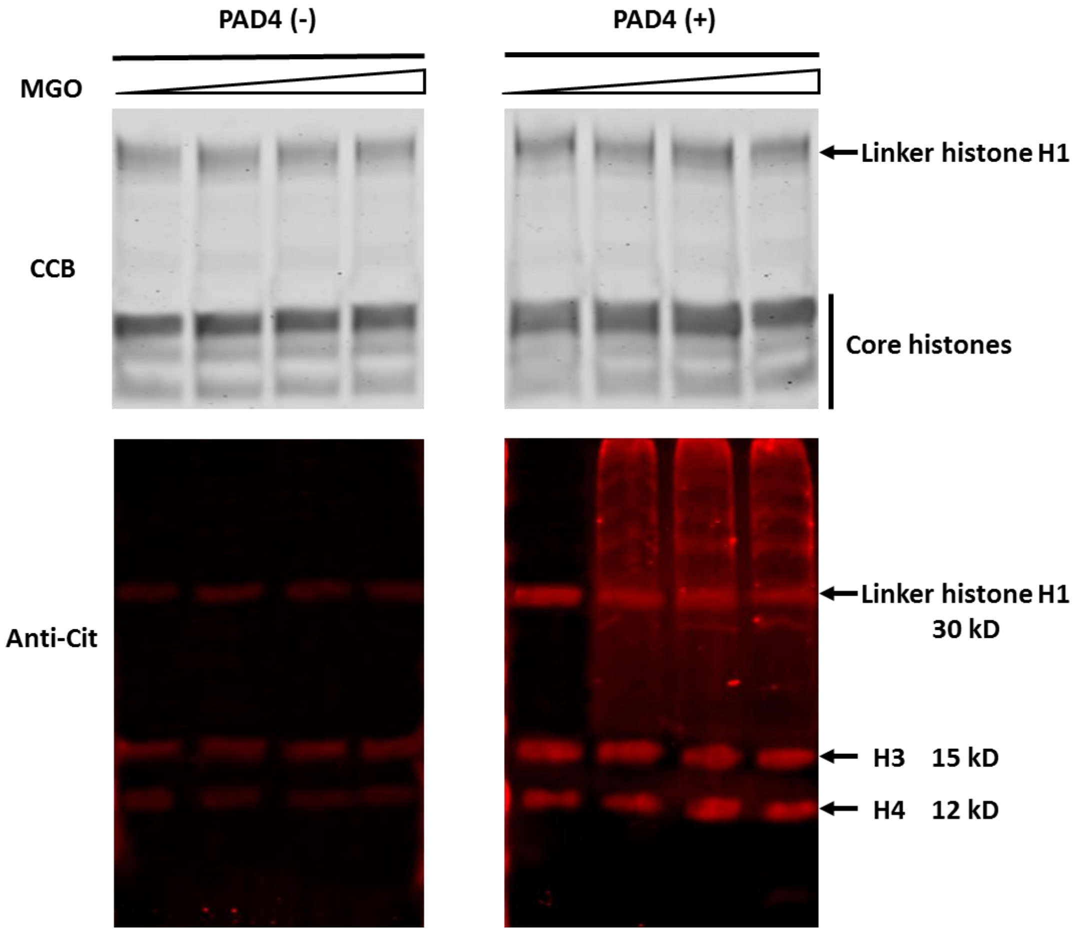

Supplementary Figure 10. Pulse-chase analysis of histone glycation and PAD4-mediated deglycation. MGO pre-treated 293T cells were transfected with (right) or without (left) pCMV-PAD4 plasmid. The histones were extracted, separated by SDS-PAGE, transferred to a PVDF membrane and blotted with indicated antibodies.

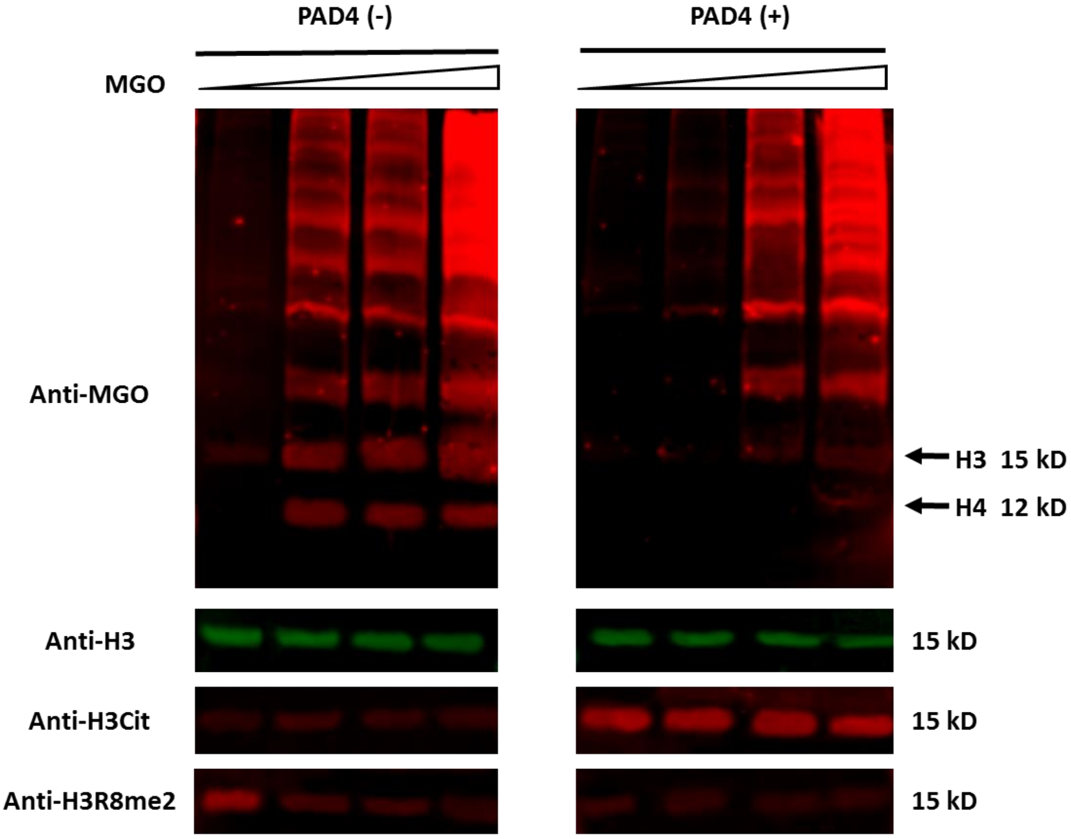

Supplementary Figure 11. Sensitivity and compaction of MNase digested chromatin from WT and PAD4-overexpressed 293T cells with (left) and without 0.25 mM MGO (right) treatment. The chromatin were isolated from the cells and then digested by MNase at room temperature for varying periods of time (0, 5, 10 and 20 min). The DNA was extracted and purified by standard procedures, and then analyzed by Tris-Borate-EDTA (TBE) gel electrophoresis.

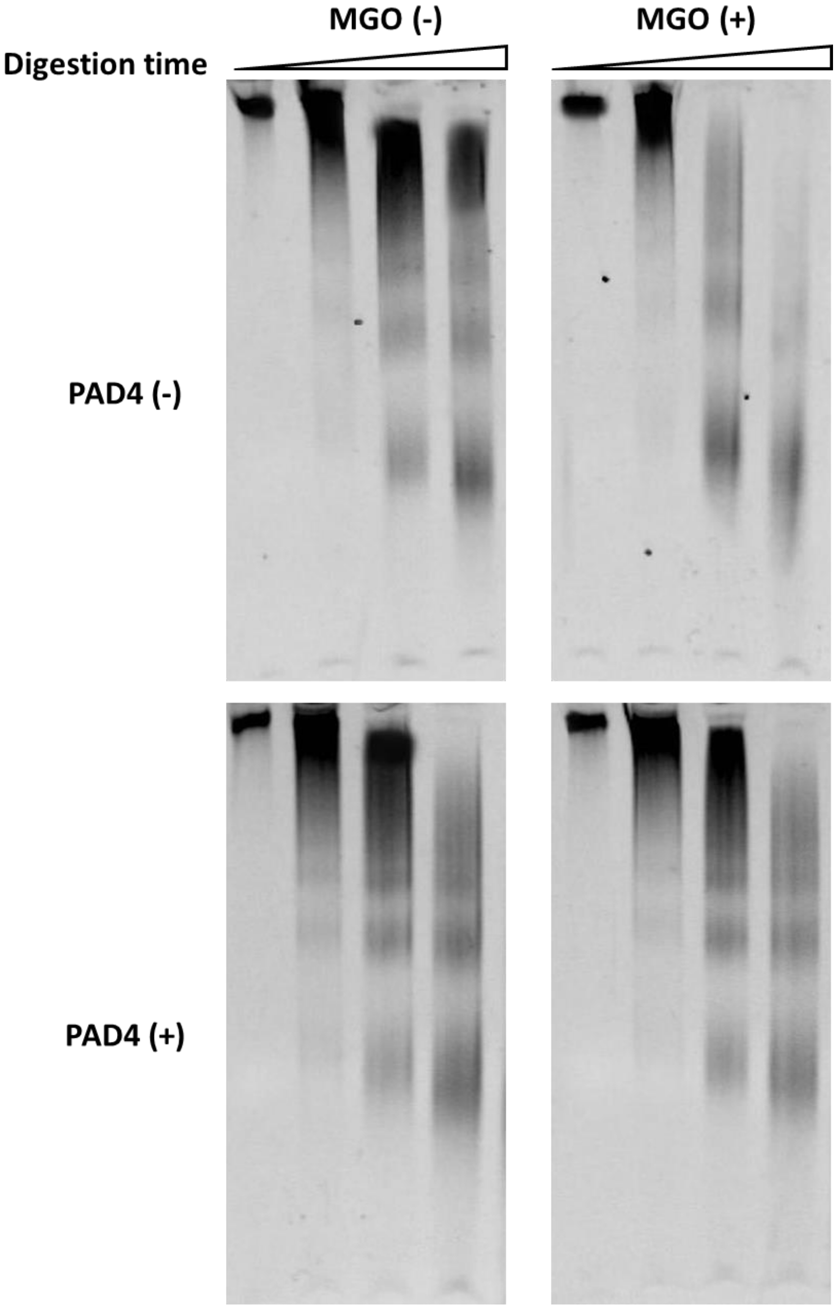

Supplementary Figure 12. Immunoprecipitation (IP) and pull-down analysis of H3 N-terminal peptide binding to catalytically dead PAD4 mutant (PAD4-C645S). (a) Either non-glycated or glycated N-terminal biotin-H3 tail (residues 1-18) was incubated with either recombinant PAD4-C645S or a 293T lysate containing overexpressed HA-PAD4-C645S at 4 °C for 2 h. Subsequently, peptides were enriched by streptavidin bead pull-down and analyzed by anti-PAD4 and anti-HA; (b) Dot blot analysis of H3 peptide MGO-glycation before and after IP.

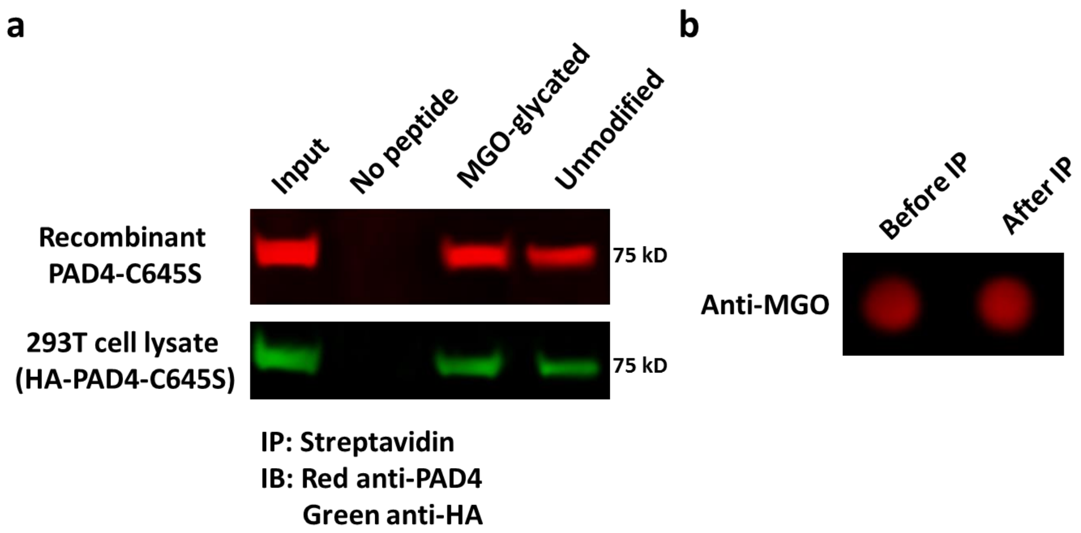

Supplementary Figure 13. Cell viability of wild type (WT) and PAD4 (or PAD4-C645S)-overexpressed 293T cells non-treated (left) or treated with gradient MGO (right). Error bars represent the standard deviation from three different experiments. Data are presented as mean values +/- SEM.

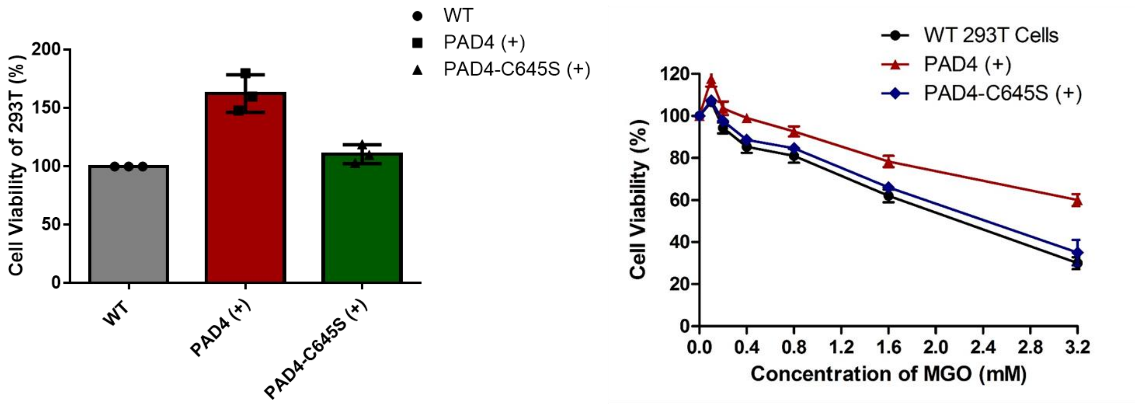

Supplementary Figure 14. PAD4 inhibitor treatment to breast cancer cell line MCF7. MCF7 cells were pre-treated with PAD4 inhibitor GSK484 and then incubated with gradient MGO (0, 0.25, 0.5 and 1.0 mM). The histones were extracted, separated by SDS-PAGE, transferred to a PVDF membrane and blotted with indicated antibodies.

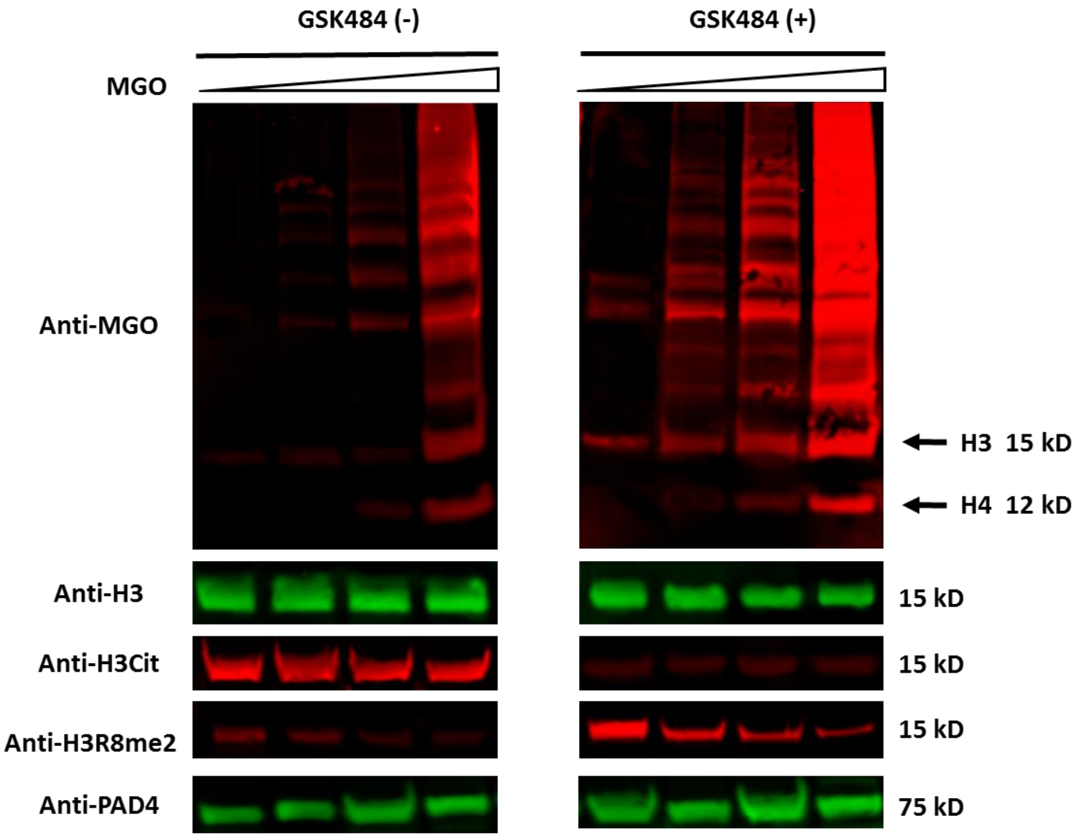

Supplementary Figure 15. Quantification of selected immunoblotting (Figures 2B, 3A and S10) in this research. The error bars represent the standard deviation from three different experiments. Error bars represent the standard deviation from three different experiments. Data are presented as mean values +/- SEM.

a

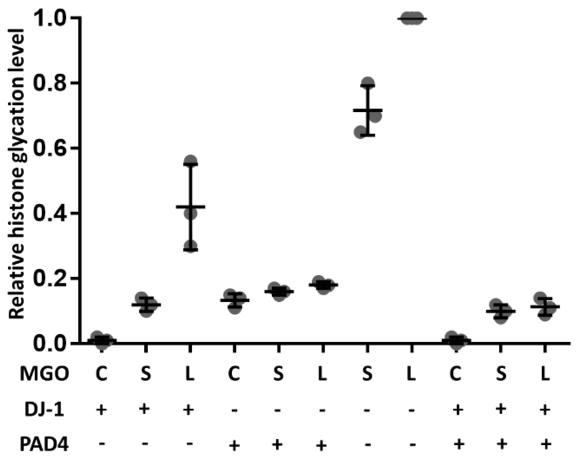

b

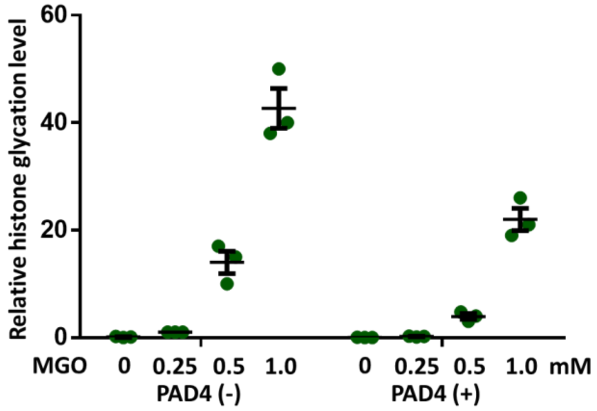

c

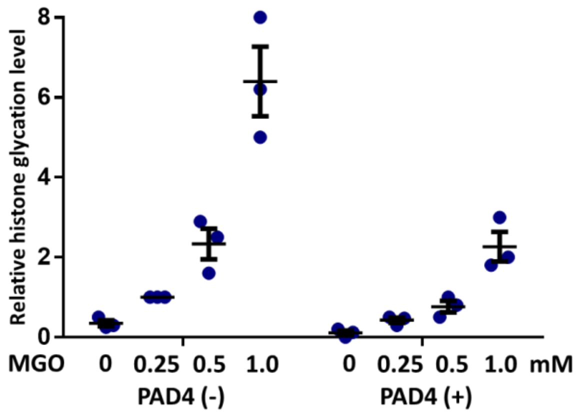

Supplementary Figure 16. Proposed mechanism of PAD4's dual function in antagonizing histone MGO-glycation: (a) protecting unmodified Arg residues via deamination and (b) rewriting MGO-modified Arg into Cit.

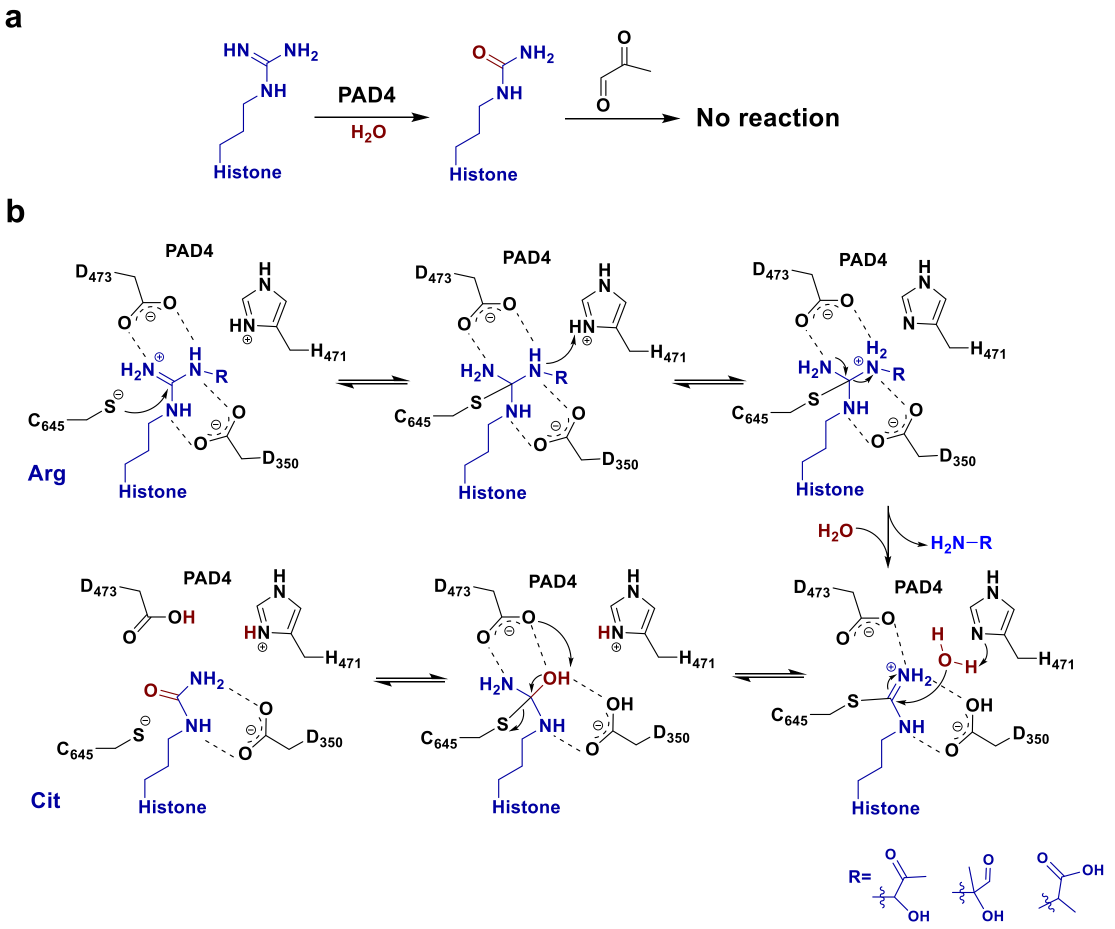

Supplementary Figure 17. Schematic of MGO-induced glycation, the resulting species that can be recognized by the Anti-MGO antibody used in this study and the PAD4 substrates.

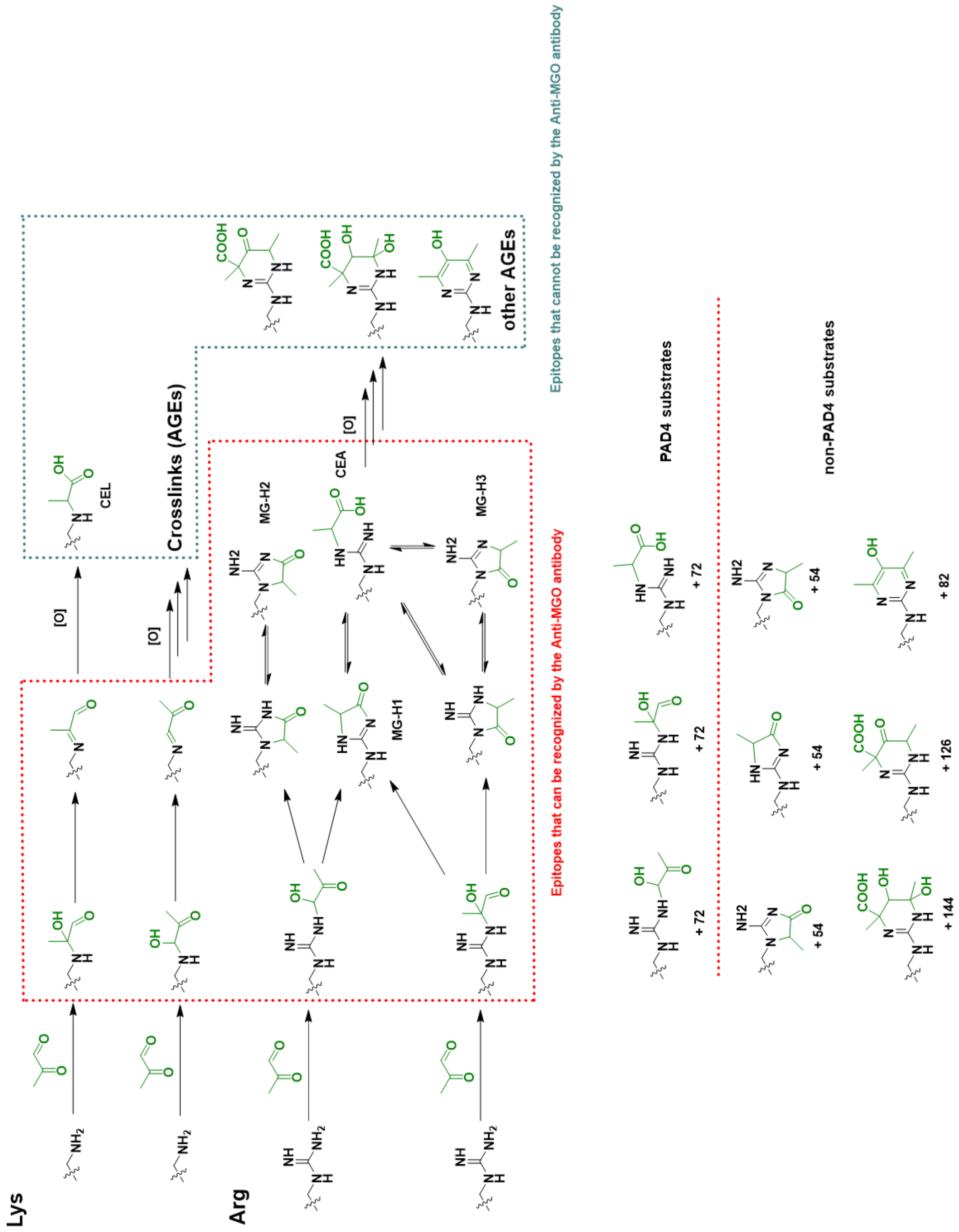

Supplementary tables

Supplementary Table 1. Primary antibodies used in this manuscript.

Host	Epitope	WB	Vendor
Mouse	Anti-MGO	1: 500	Cell Biolabs (STA-011)
Chicken	Anti-H3	1: 1000	Abcam (ab134198)
Mouse	Anti-H3	1: 1000	Abcam (ab10799)
Rabbit	Anti-H3Cit (R2, R8, R17)	1: 1000	Abcam (ab5103)
Rabbit	Anti-PAD4	1: 1000	Abcam (ab50332)
Rabbit	Anti-HA	1:1000	CST (3724S)
Mouse	Anti-Actin	1: 1000	CST (3700S)
Rabbit	Anti-H3R8Me2	1: 1000	Abcam (ab194692)
Mouse	Anti-Citrulline	1:1000	Sigma (SAB5202274)
Chicken	Anti-H4	1:1000	Abcam (ab134212)
Rabbit	Anti-H4Cit3	1:1000	MilliporeSigma (07-596)
Rabbit	Anti-H4R3me2	1:1000	Abcam (ab194683)

Supplementary Table 2. Secondary antibodies used in this manuscript.

Host	Epitope	Label	Dilution	Vendor
Donkey	Anti-Chicken	IRDye 800CW	1: 15000	Li-Cor
Goat	Anti-Mouse	IRDye 680RD	1: 15000	Li-Cor
Goat	Anti-Mouse	IRDye 800CW	1: 15000	Li-Cor
Goat	Anti-Rabbit	IRDye 800CW	1: 15000	Li-Cor
Goat	Anti-Rabbit	IRDye 680RD	1: 15000	Li-Cor
-	Biotin	Atto 680-Streptavidin	1:20000	Sigma
