# Supplementary material for: Protein arginine deiminase 4 antagonizes methylglyoxal-induced histone glycation
Source: Nat Commun. 2020 Jun 26;11:3241. doi: 10.1038/s41467-020-17066-y (PMC7319962; doi:10.1038/s41467-020-17066-y)
Supplement: Supplementary file 3 — Reporting Summary [file 41467_2020_17066_MOESM3_ESM.pdf]

nature research

Corresponding author(s): Yael David

Last updated by author(s): May 26, 2020

Reporting Summary

Nature Research wishes to improve the reproducibility of the work that we publish. This form provides structure for consistency and transparency in reporting. For further information on Nature Research policies, see Authors & Referees and the Editorial Policy Checklist .

Statistics

For all statistical analyses, confirm that the following items are present in the figure legend, table legend, main text, or Methods section.

n/a	Confirmed	
☐	☒	The exact sample size (*n*) for each experimental group/condition, given as a discrete number and unit of measurement
☐	☒	A statement on whether measurements were taken from distinct samples or whether the same sample was measured repeatedly
☒	☐	The statistical test(s) used AND whether they are one- or two-sided<br>*Only common tests should be described solely by name; describe more complex techniques in the Methods section.*
☒	☐	A description of all covariates tested
☒	☐	A description of any assumptions or corrections, such as tests of normality and adjustment for multiple comparisons
☐	☒	A full description of the statistical parameters including central tendency (e.g. means) or other basic estimates (e.g. regression coefficient) AND variation (e.g. standard deviation) or associated estimates of uncertainty (e.g. confidence intervals)
☒	☐	For null hypothesis testing, the test statistic (e.g. *F*, *t*, *r*) with confidence intervals, effect sizes, degrees of freedom and *P* value noted<br>*Give P values as exact values whenever suitable.*
☒	☐	For Bayesian analysis, information on the choice of priors and Markov chain Monte Carlo settings
☒	☐	For hierarchical and complex designs, identification of the appropriate level for tests and full reporting of outcomes
☒	☐	Estimates of effect sizes (e.g. Cohen's *d*, Pearson's *r*), indicating how they were calculated

Our web collection on statistics for biologists contains articles on many of the points above.

Software and code

Policy information about availability of computer code

Data collection	No special software was used for data collection.
Data analysis	Microsoft Excel, GraphPad Prism 7

For manuscripts utilizing custom algorithms or software that are central to the research but not yet described in published literature, software must be made available to editors/reviewers. We strongly encourage code deposition in a community repository (e.g. GitHub). See the Nature Research guidelines for submitting code & software for further information.

Data

Policy information about availability of data

All manuscripts must include a data availability statement. This statement should provide the following information, where applicable:

- Accession codes, unique identifiers, or web links for publicly available datasets
- A list of figures that have associated raw data
- A description of any restrictions on data availability

All the data are available within the article and its Supplementary Information files or from the corresponding author upon reasonable request. A reporting summary for this article is available as a Supplementary Information file. The source data underlying Figures 2, 3 and Supplementary Figures 2, 3, 4, 7, 8, 10, 12, 13, 14, 15 are provided as a Source Data file.

Field-specific reporting

Please select the one below that is the best fit for your research. If you are not sure, read the appropriate sections before making your selection.

Life sciences study design

All studies must disclose on these points even when the disclosure is negative.

Sample size	Sample size was chosen based on our previous work using the same samples and models (Nat. Commun., 2019, 10: 1289).
Data exclusions	No data was excluded from the analysis.
Replication	All the experiments in this research were repeated independently three times with similar results.
Randomization	All the samples were allocated into experimental groups randomly. Animals were randomized according to weight at the start of studies.
Blinding	Blinding was not necessary as we were observing large differences in staining or morphology.

Reporting for specific materials, systems and methods

We require information from authors about some types of materials, experimental systems and methods used in many studies. Here, indicate whether each material, system or method listed is relevant to your study. If you are not sure if a list item applies to your research, read the appropriate section before selecting a response.

Materials & experimental systems

n/a	Involved in the study
☐	☒ Antibodies
☐	☒ Eukaryotic cell lines
☒	☐ Palaeontology
☐	☒ Animals and other organisms
☐	☒ Human research participants
☒	☐ Clinical data

Methods

n/a	Involved in the study
☒	☐ ChIP-seq
☒	☐ Flow cytometry
☒	☐ MRI-based neuroimaging

Antibodies

Antibodies used	Mouse Anti-Methylglyoxal Monoclonal Antibody (Cell Biolabs, STA-011), Chicken Anti-H3 Polyclonal Antibody (Abcam, ab134198), Mouse Anti-H3 Monoclonal Antibody (Abcam, ab10799), Mouse Anti-Actin Monoclonal Antibody (CST, 3700S), Rabbit Anti-H3R8Me2 Polyclonal Antibody (Abcam, ab194692), Rabbit Anti-H3Cit (R2, R8, R17) Polyclonal Antibody (Abcam, ab5103), Mouse Anti-Citrulline Monoclonal Antibody (Sigma, SAB5202274), Rabbit Anti-PAD4 Polyclonal Antibody (Abcam, ab50332), Chicken Anti-H4 Polyclonal Antibody (Abcam, ab134212), Rabbit Anti-H4Cit3 Polyclonal Antibody (MilliporeSigma, 07-596), Rabbit Anti-H4R3me2 Polyclonal Antibody (Abcam, ab194683), Rabbit Anti-HA Monoclonal Antibody (CST, 3724S), Rabbit Anti-MEK ½ Polyclonal Antibody (CST, 9122S). All the secondary antibodies (listed in Supplementary Table 2) used in this manuscript are obtained from Li-Cor.
Validation	Each primary antibody used in this study was validated for species (human or mouse) and application (immunblotting and IP) by the above-listed manufacturer using recombinant protein and/or expressing cell types as a positive control; these validation studies are reported for each primary antibody on the product website. In addition, primary antibodies were validated for species and application (immunblotting and IP) in our experiments using recombinant proteins and/or expressing cell types as a positive control and are reported in main text and supplemental figures (see our previous work Nat. Commun., 2019, 10: 1289). For more validation data of the antibodies used in this study, see the product information of the corresponding suppliers' websites.

Eukaryotic cell lines

Policy information about cell lines

Cell line source(s)	MCF7 is obtained from DSMZ. HEK293T, T47D, BT474, ZR75-1, and Cama-1 cell lines used in this study are all sourced from ATCC.
Authentication	All cell lines used in this manuscript were authenticated by the vendors where we acquired these cell lines. ATCC used morphology, kryotyping, and PCR-based approaches.
Mycoplasma contamination	All cell lines were tested for mycoplasma monthly (MycoFluor Mycoplasma detection kit, Invitrogen) and tested negative for contamination.
Commonly misidentified lines (See ICLAC register)	No commonly misidentified cell lines were used in this study.

Animals and other organisms

Policy information about studies involving animals; ARRIVE guidelines recommended for reporting animal research

Laboratory animals	Six-to-8-week-old nu/nu athymic BALB/c female mice (Harlan Laboratories, Inc.) were used for the breast cancer xenografts experiments and maintained in pressurized ventilated caging. All these studies were performed in compliance with institutional guidelines under an Institutional Animal Care and Use Committee-approved protocol (MSKCC#12–10–016).
Wild animals	The study did not involve any wild animals.
Field-collected samples	The study did not involve any samples collected from the field.
Ethics oversight	All the animal studies were performed in compliance with institutional guidelines under an Institutional Animal Care and Use Committee-approved protocol (MSKCC#12–10–016).

Note that full information on the approval of the study protocol must also be provided in the manuscript.

Human research participants

Policy information about studies involving human research participants

Population characteristics	N/A
Recruitment	N/A
Ethics oversight	The clinical samples (MSKCC set) used in this study were obtained from the Biobank of MSKCC. The patients with breast cancer and either recurrence of disease after receiving adjuvant therapy or WHO-defined progression of metastatic disease on therapy were prospectively enrolled on an IRB approved tissue collection protocol (IRB#06-163).

Note that full information on the approval of the study protocol must also be provided in the manuscript.
