# Supplementary material for: Protein arginine deiminase 4 antagonizes methylglyoxal-induced histone glycation
Source: Nat Commun. 2020 Jun 26;11:3241. doi: 10.1038/s41467-020-17066-y (PMC7319962; doi:10.1038/s41467-020-17066-y)
Supplement: Supplementary file 4 — Source Data [file 41467_2020_17066_MOESM4_ESM.pdf]

Figure 2

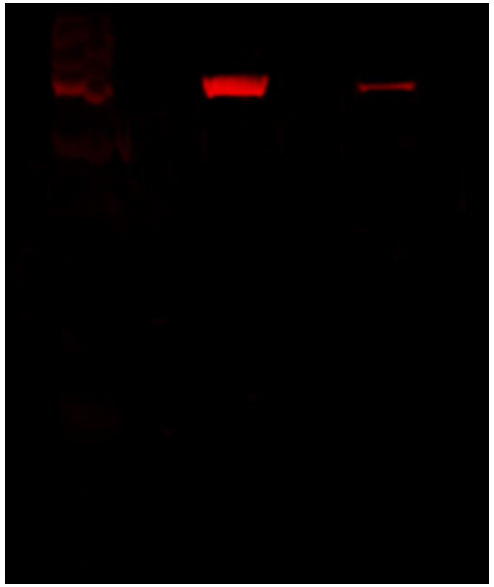

Fig. 2a (anti-MGO)

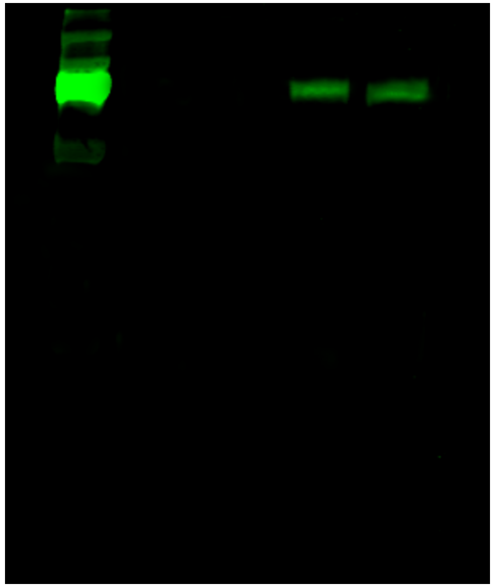

Fig. 2a (anti-H3 Cit)

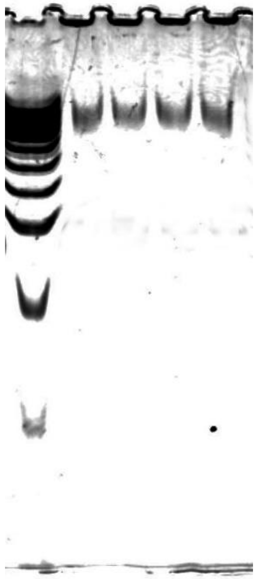

Fig. 2a (EtBr)

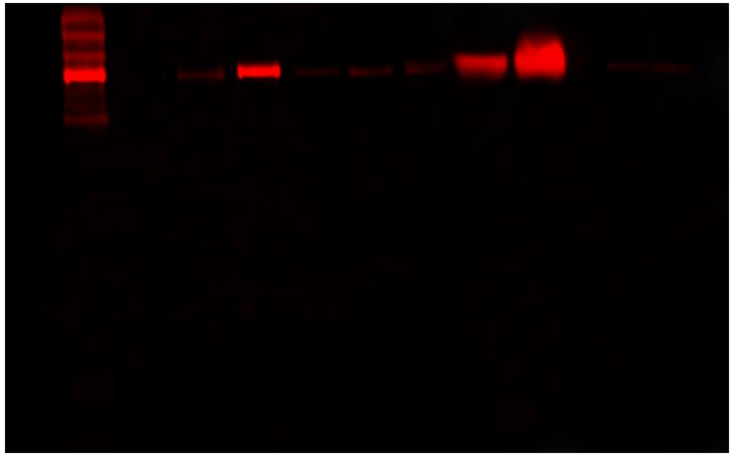

Fig. 2b (anti-MGO)

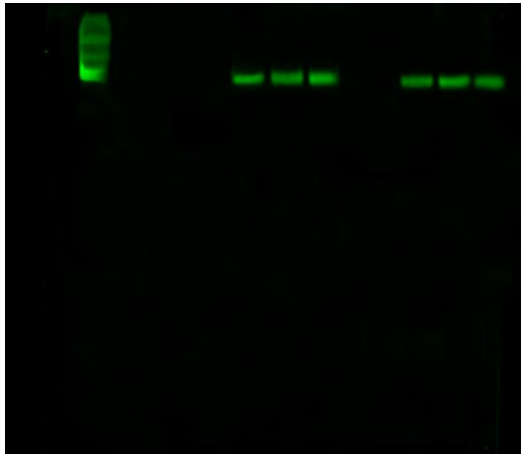

Fig. 2b (anti-H3 Cit)

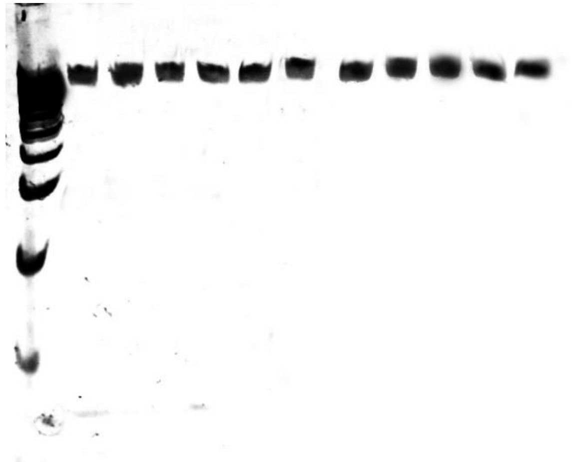

Fig. 2b (EtBr)

Figure 2

	MGO -, PAD4 -			MGO +, PAD4 -			MGO -, PAD4 +			MGO +, PAD4 + (C)			MGO + (S), PAD4 +			MGO + (L), PAD4 +		
0.0	100.0	100.0	100.0	100.0	100.0	100.	100.0	100.0	100.0	100.0	100.0	100.0	100.0	100.0	100.0	100.0	100.0	100.0
0.5	83.0	82.0	78.0	98.0	96.0	100.	83.0	87.0	90.0	88.0	92.0	94.0	92.0	95.0	95.0	93.0	96.0	98.0
1.0	75.9	70.0	64.0	95.0	93.0	86.	78.0	75.0	71.0	81.0	78.0	75.0	84.0	82.0	78.0	84.0	85.0	86.0
1.5	62.0	50.0	64.0	89.5	82.0	90.	70.0	65.0	63.0	73.0	68.0	66.0	77.0	74.0	70.0	80.0	75.0	76.0
2.0	47.0	44.5	30.3	68.0	75.0	79.	46.0	55.0	54.0	49.0	60.0	59.0	53.0	62.0	64.0	56.0	68.0	65.0
2.5	10.1	14.0	5.6	58.0	40.0	53.	28.0	20.0	22.0	30.0	23.0	25.0	34.0	25.0	28.0	37.0	28.0	32.0

Fig. 2c

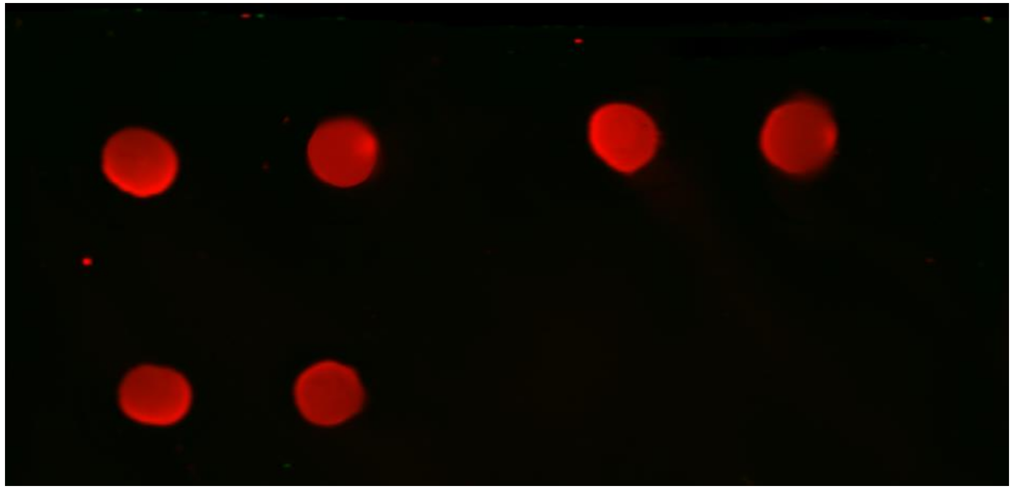

Atto 680-Streptavidin

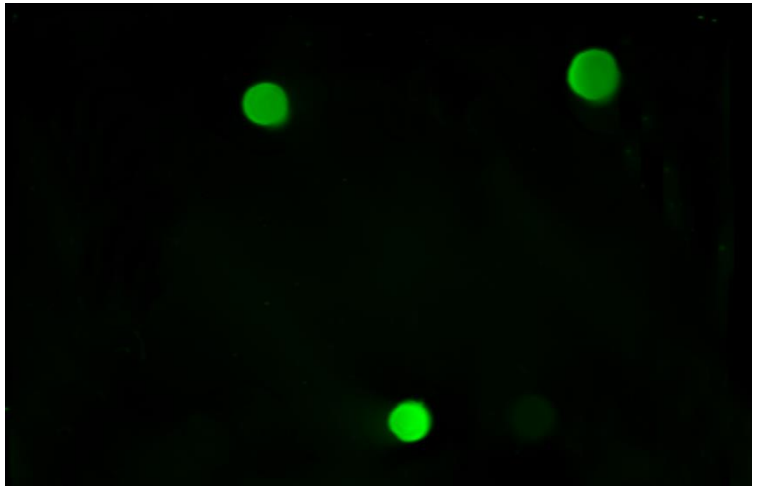

anti-MGO

Fig. 2d

Figure 3

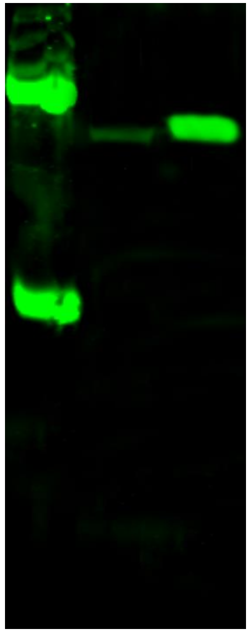

Fig. 3a (anti-PAD4)

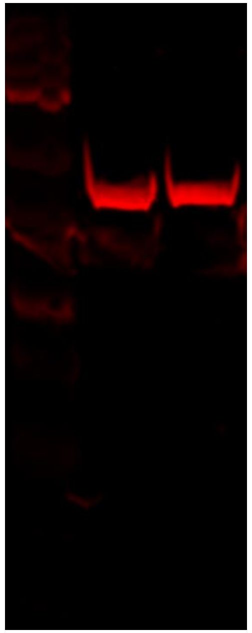

Fig. 3a (anti-actin)

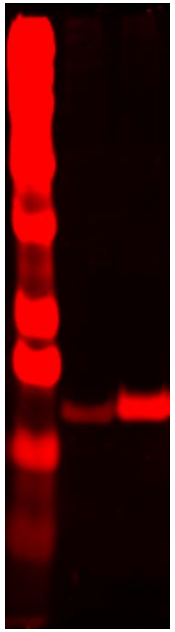

Fig. 3a (anti-H3Cit)

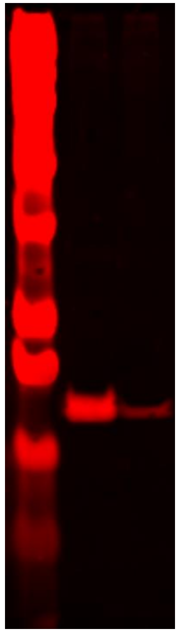

Fig. 3a (anti-H3R8me2)

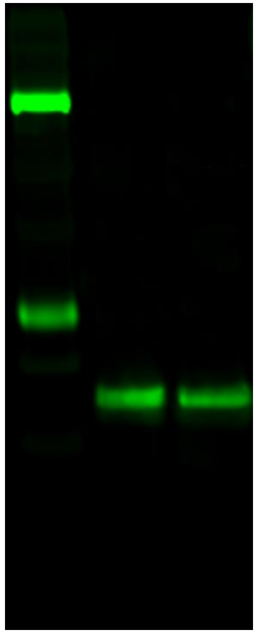

Fig. 3a (anti-H3)

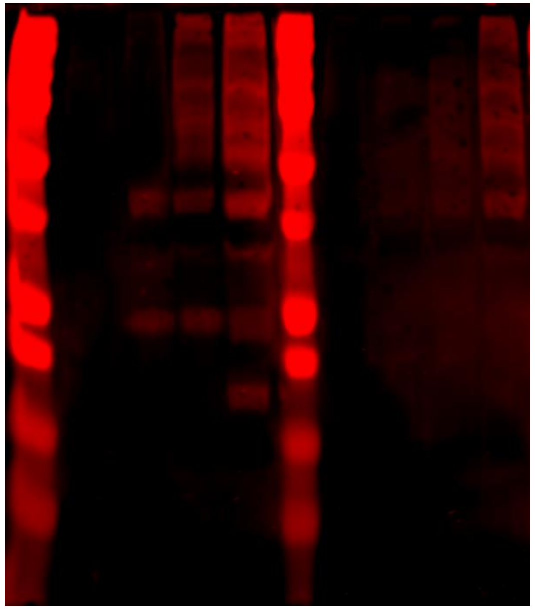

Fig. 3a (anti-MGO)

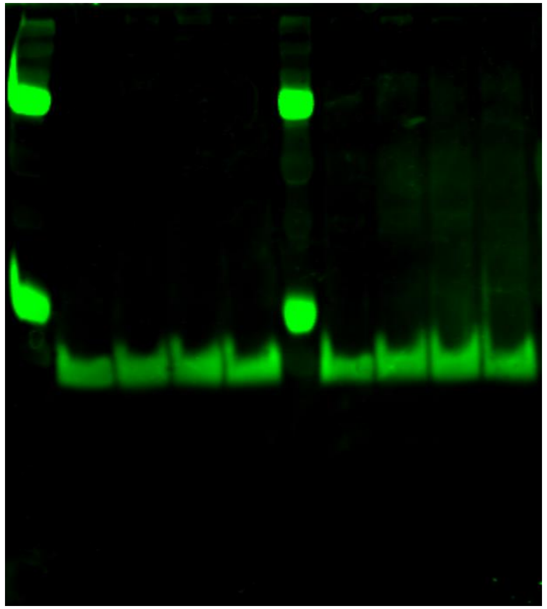

Fig. 3a (anti-H3)

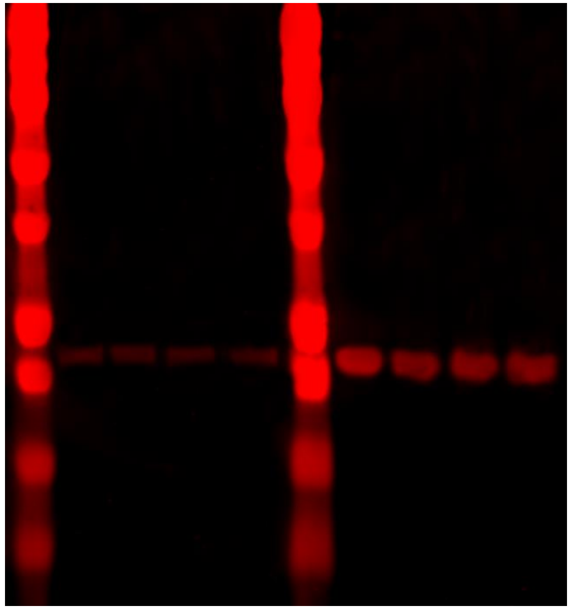

Fig. 3a (anti-H3Cit)

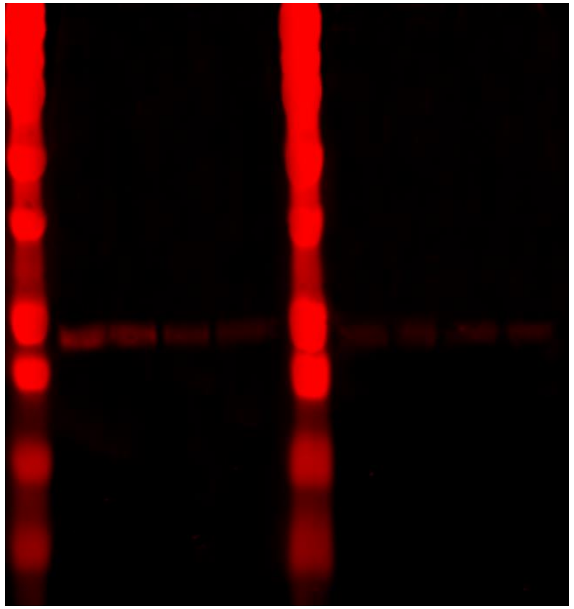

Fig. 3a (anti-H3R8me2)

Figure 3

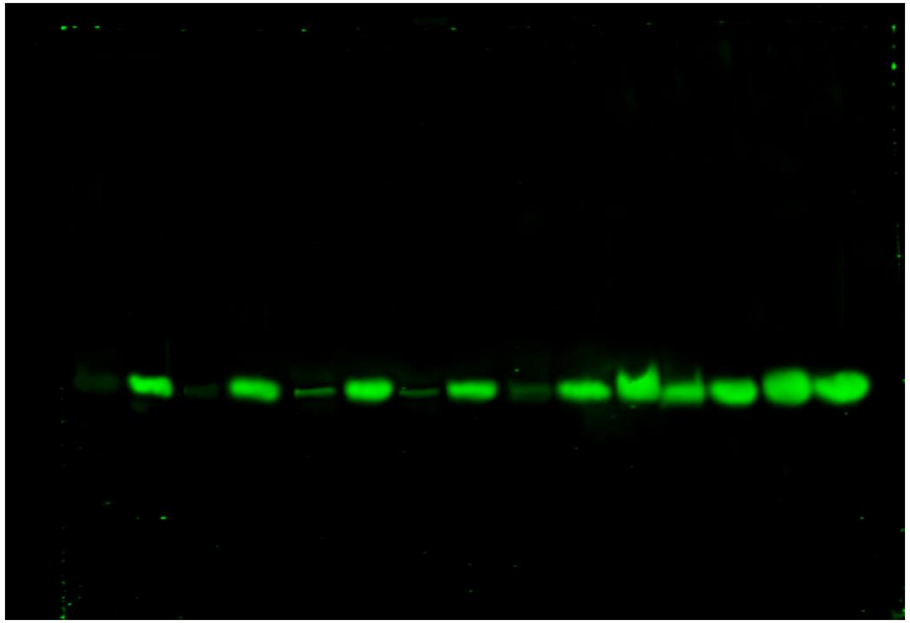

Fig. 3b (anti-H3)

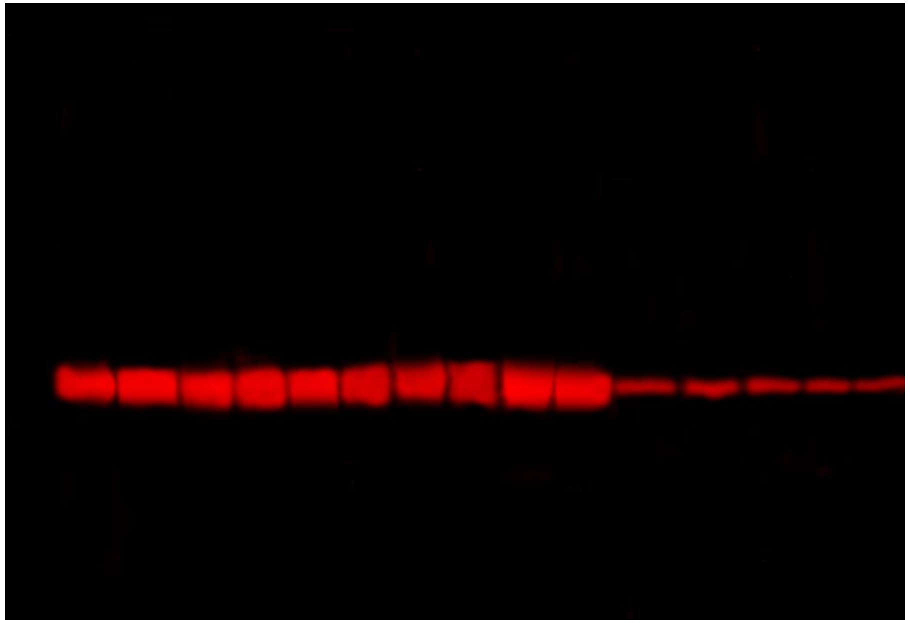

Fig. 3b (anti-H3Cit)

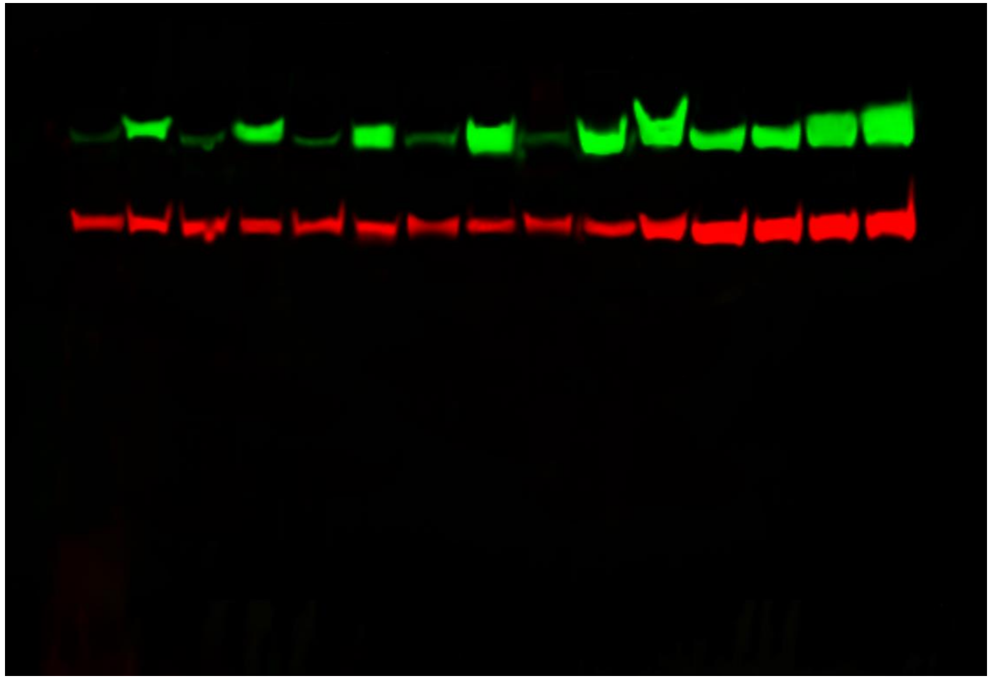

Fig. 3b (anti-PAD4 & anti-actin)

Supplementary Figure 2

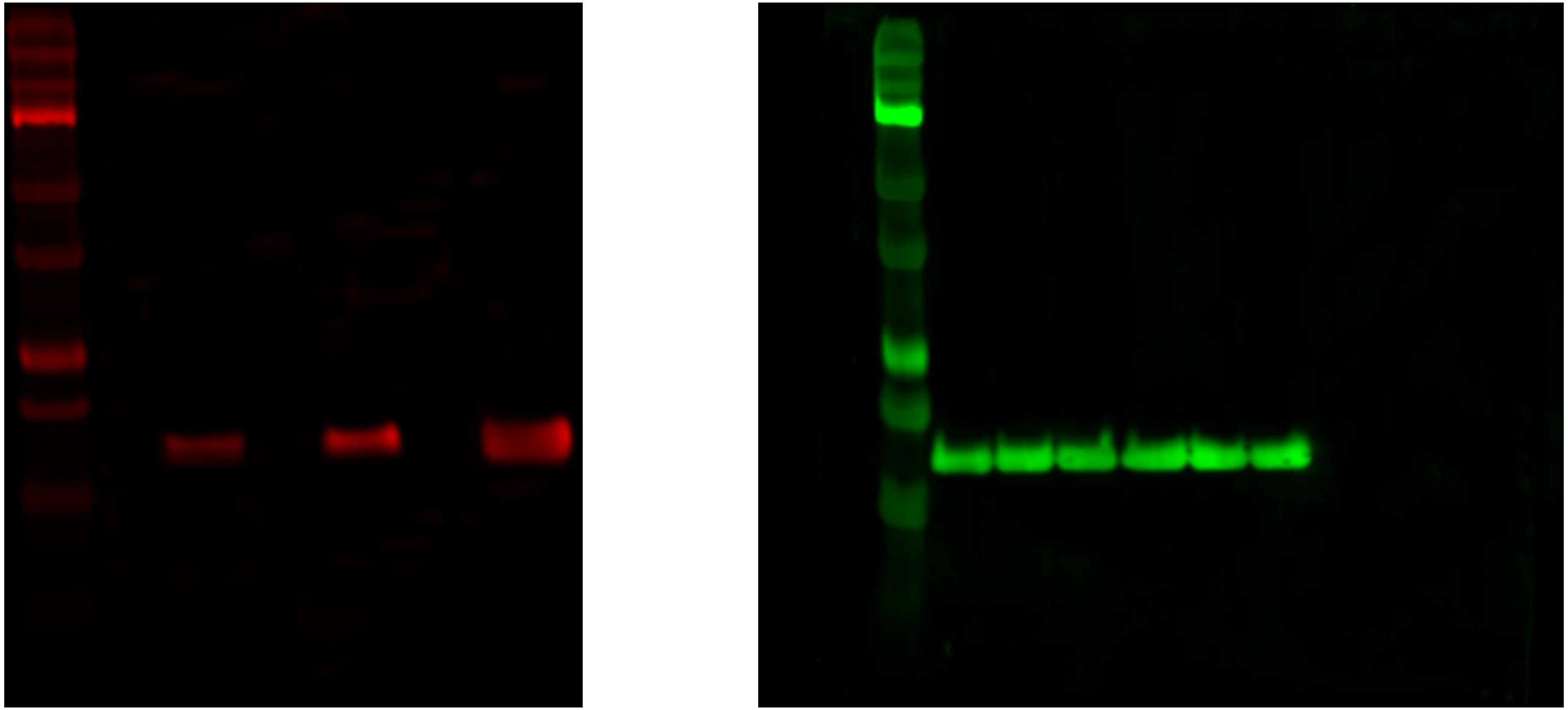

anti-H3Cit

anti-H3

Supplementary Figure 3

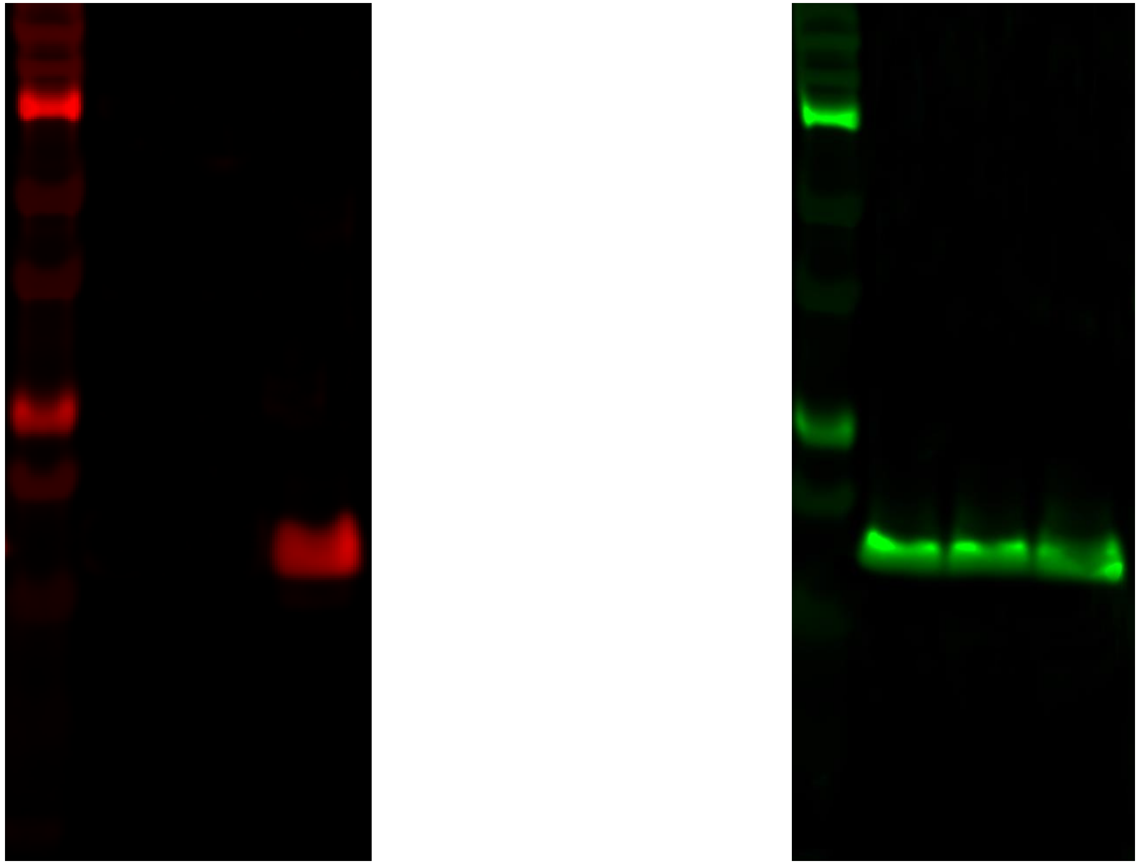

anti-H3Cit

anti-H3

Supplementary Figure 4

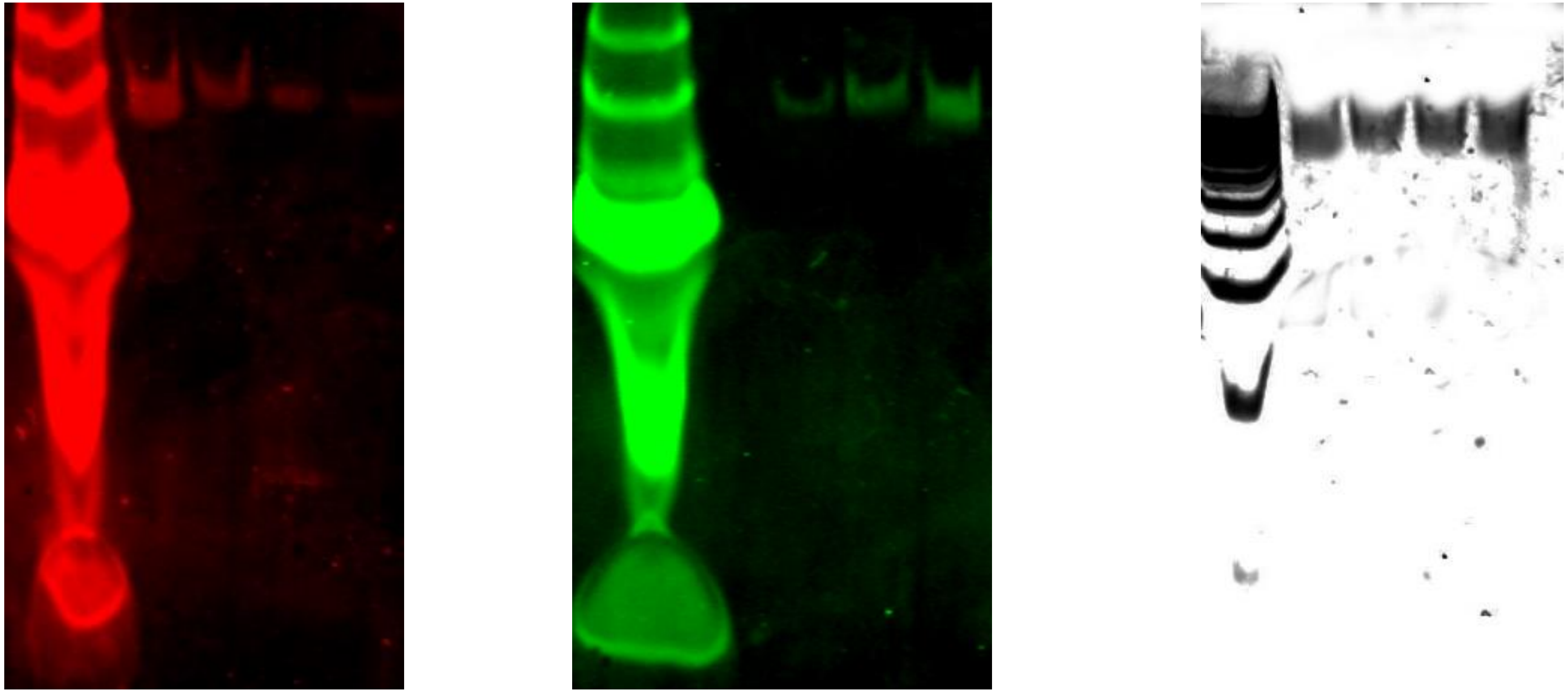

anti-MGO anti-H3Cit EtBr

Supplementary Figure 7

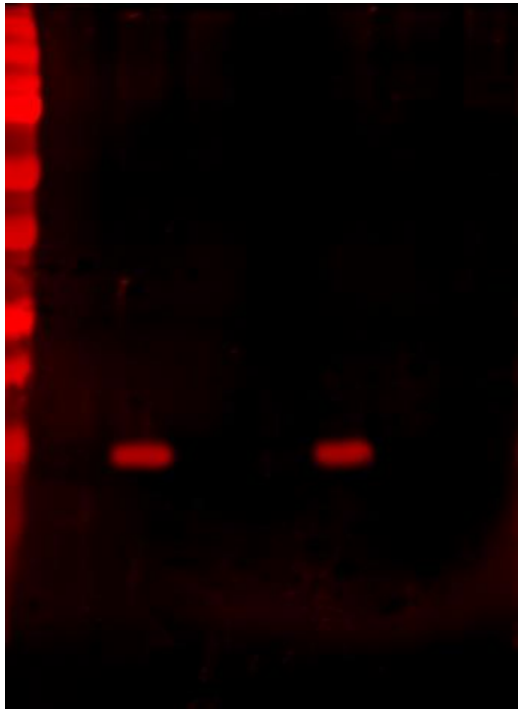

anti-H4Cit3

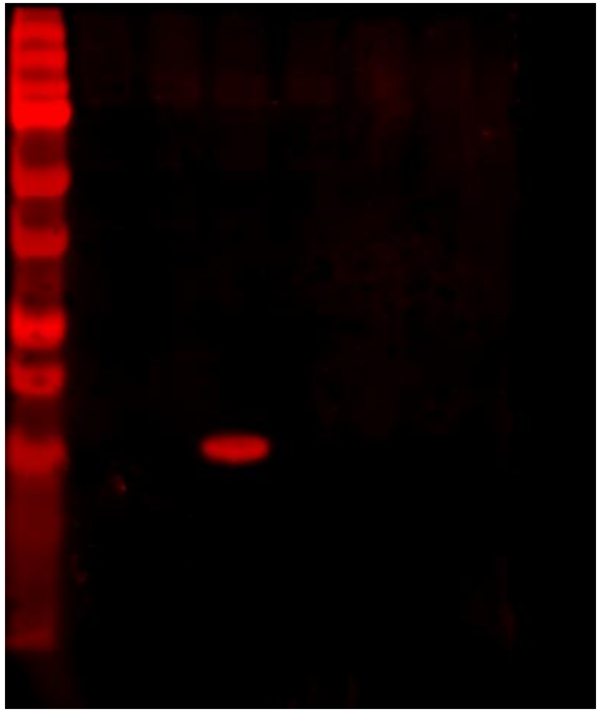

anti-H4R3me2

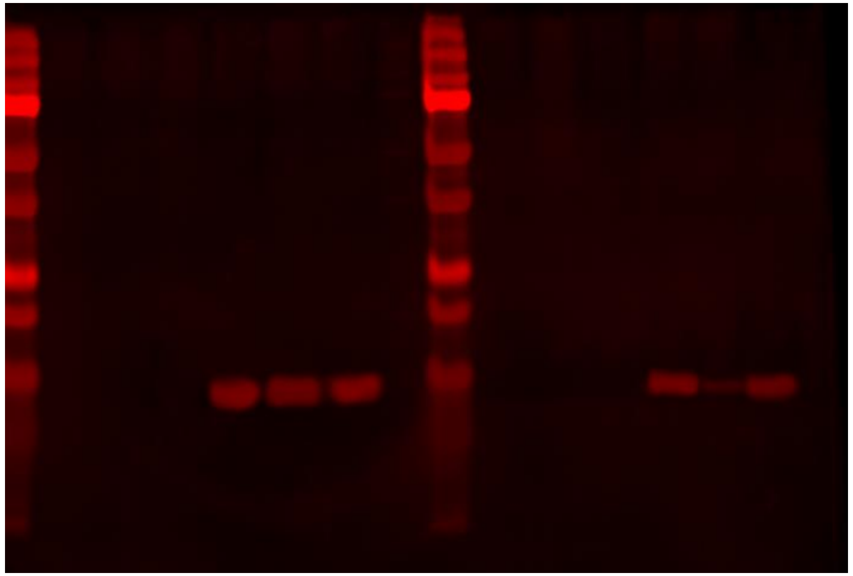

anti-MGO

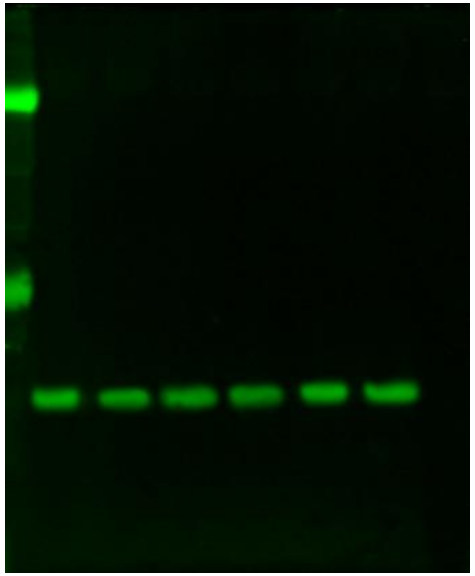

anti-H4

Supplementary Figure 8

	MGO -, DJ-1 -			MGO +, DJ-1 -			MGO -, DJ-1 +			MGO +, DJ-1 + (Co)			MGO + (S), DJ-1 +			MGO + (L), DJ-1 +		
0.0	100.0	100.0	100.0	100.0	100.0	100.	100.0	100.0	100.0	100.0	100.0	100.0	100.0	100.0	100.0	100.0	100.0	100.0
0.5	83.0	82.0	78.0	98.0	96.0	100.	80.0	79.0	74.0	84.0	83.0	80.0	88.0	90.0	84.0	96.0	97.0	96.0
1.0	75.9	70.0	64.0	95.0	93.0	86.	73.0	68.0	60.0	76.0	72.0	65.0	82.0	74.0	68.0	90.0	88.0	89.0
1.5	62.0	50.0	64.0	89.5	82.0	90.	59.0	48.0	62.0	63.0	51.0	65.0	68.0	55.0	66.0	86.0	82.0	84.0
2.0	47.0	44.5	30.3	68.0	75.0	79.	45.0	41.0	29.0	48.0	46.0	32.0	50.0	53.0	39.0	62.0	68.0	70.0
2.5	10.1	14.0	5.6	58.0	40.0	53.	9.0	10.0	5.3	11.0	15.0	7.0	14.0	12.0	16.0	47.0	36.0	49.0

Fig. S8a

	MGO -, enzyme -			MGO +, enzyme -			MGO +, PAD4 +, DJ1 +			MGO +, mutant enzyme +		
0.0	100.0	100.0	100.0	100.0	100.0	100.	100.0	100.0	100.0	100.0	100.0	100.0
0.5	83.0	82.0	78.0	98.0	96.0	100.	83.0	87.0	90.0	99.0	100.0	98.0
1.0	75.9	70.0	64.0	95.0	93.0	86.	78.0	75.0	71.0	92.0	93.0	89.0
1.5	62.0	50.0	64.0	89.5	82.0	90.	70.0	65.0	63.0	82.0	87.0	88.0
2.0	47.0	44.5	30.3	68.0	75.0	79.	46.0	55.0	54.0	64.0	70.0	82.0
2.5	10.1	14.0	5.6	58.0	40.0	53.	28.0	20.0	22.0	55.0	48.0	56.0

Fig. S8b

Supplementary Figure 10

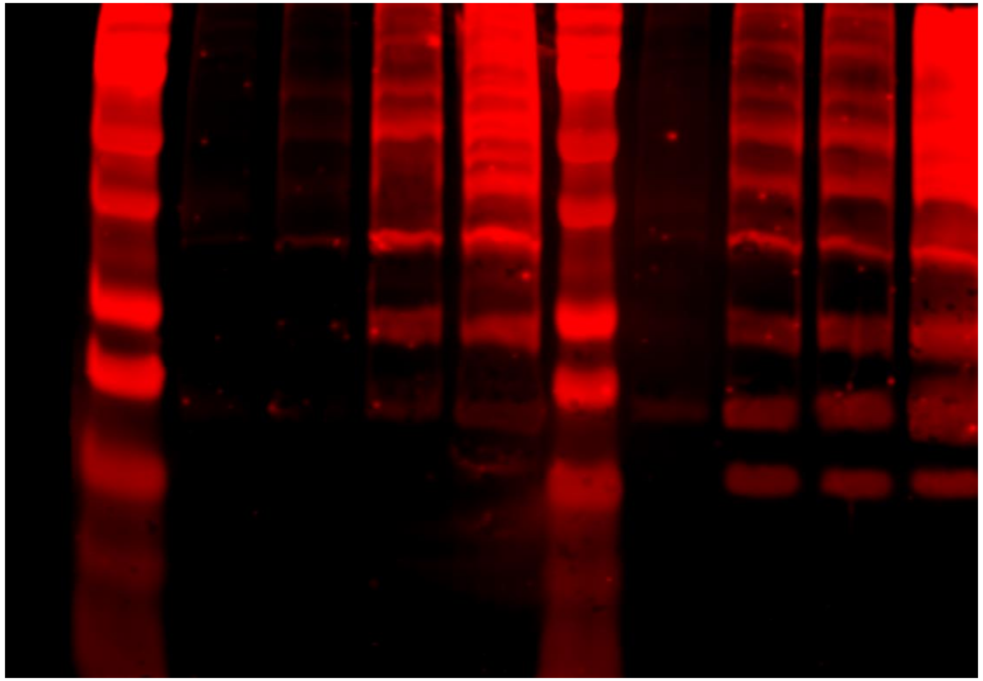

anti-MGO

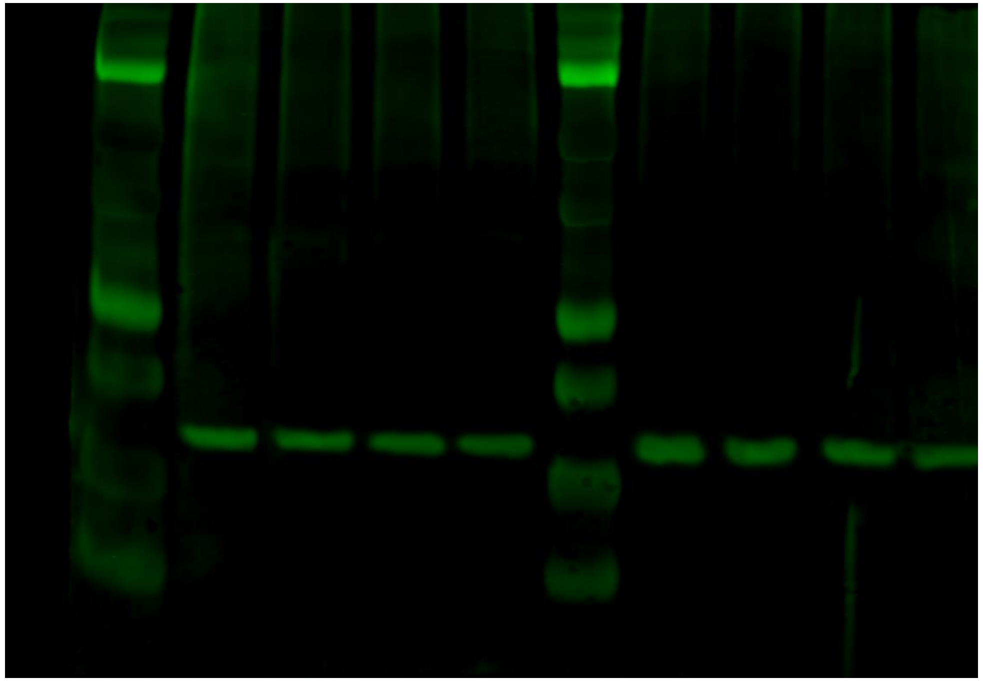

anti-H3

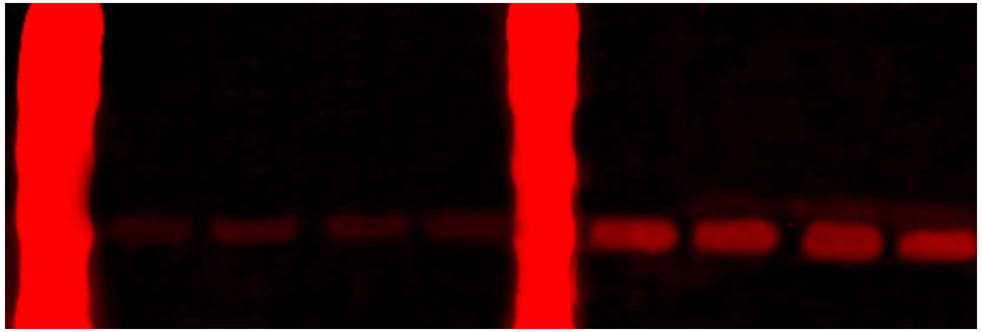

anti-H3Cit

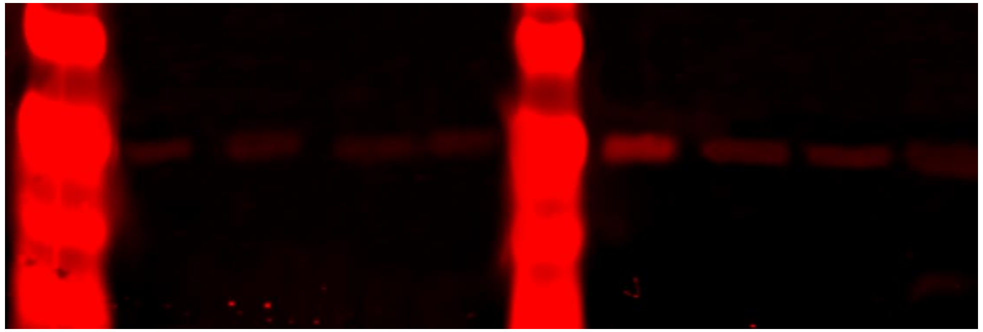

anti-H3R8me2

Supplementary Figure 12

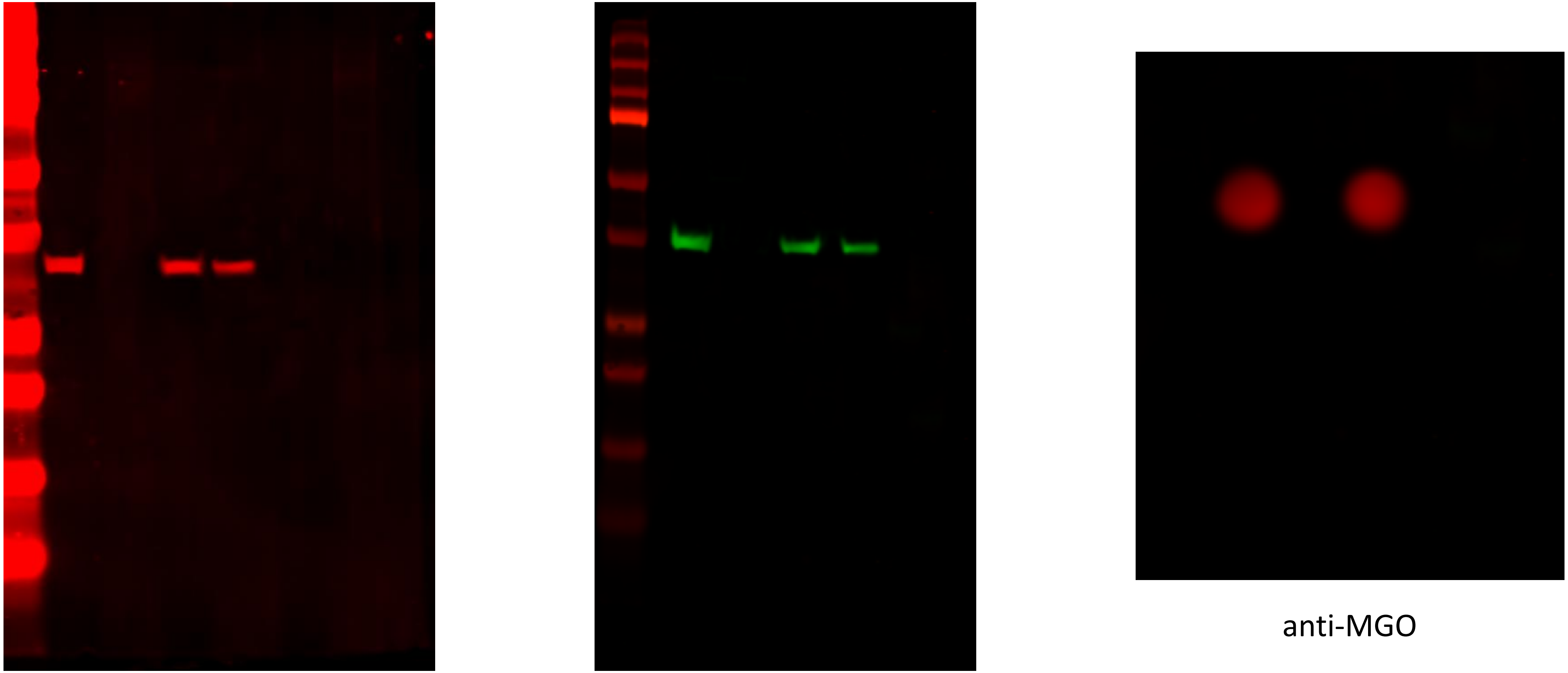

anti-PAD4 anti-HA anti-MGO

Supplementary Figure 13

WT	PAD4 (+)	PAD4-C645S (+)
100.	160.	103.
100.	180.	110.
100.	148.	119.

	WT 293T Cells			PAD4 (+)			PAD4-C645S (+)		
0.0	100.	100.	100.	100.	100.	100.	100.	100.	100.
0.1	106.	104.	110.	111.	118.	124.	106.	107.	109.
0.2	94.	99.	90.	100.	110.	101.	98.	99.	96.
0.4	90.	80.	86.	102.	96.	99.	92.	86.	88.
0.8	82.	75.	86.	96.	88.	94.	87.	81.	86.
1.6	58.	60.	68.	82.	73.	80.	64.	65.	69.
3.2	25.	30.	35.	55.	65.	60.	24.	36.	45.

Supplementary Figure 14

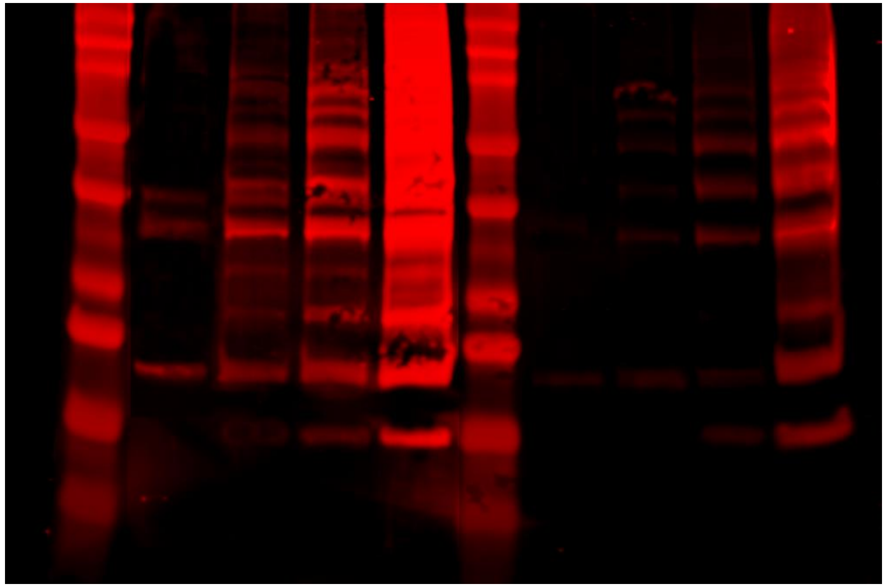

anti-MGO

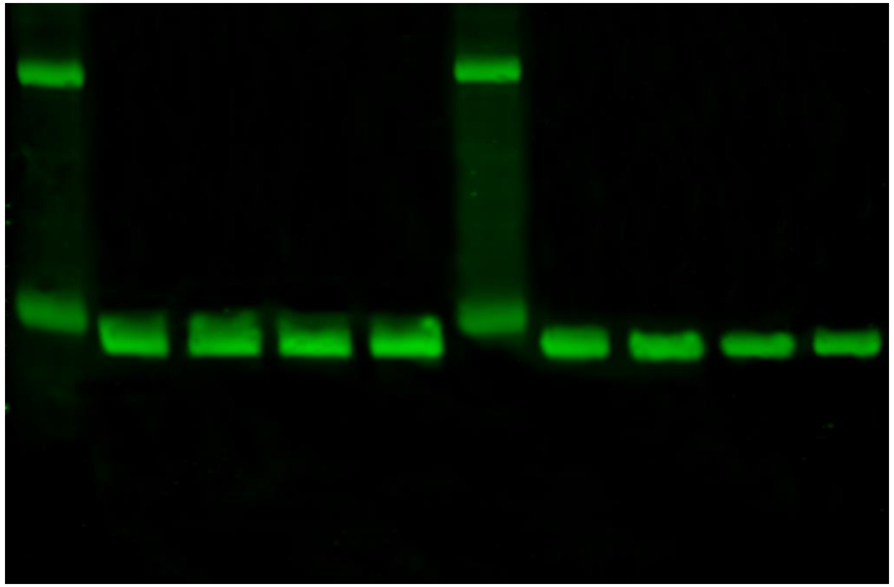

anti-H3

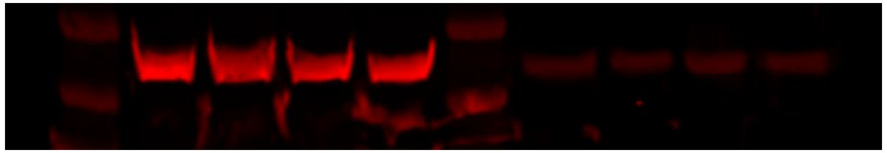

anti-H3Cit

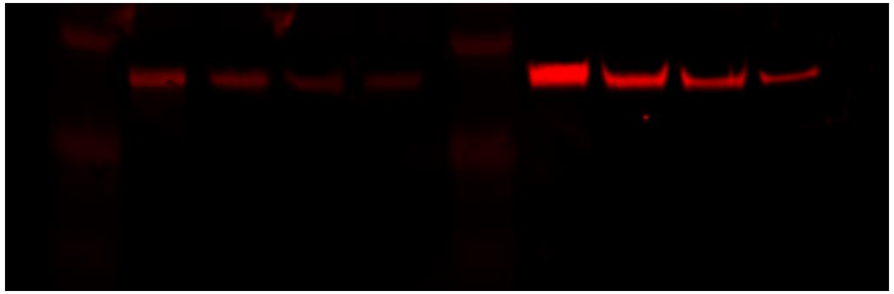

anti-H3R8me2

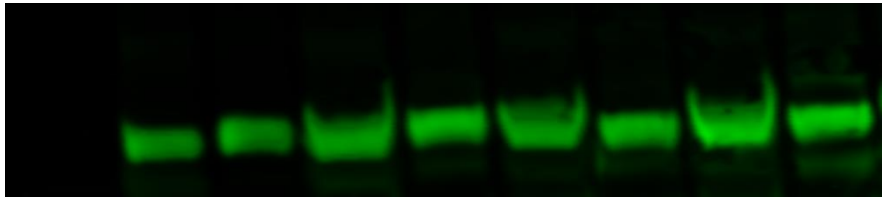

anti-PAD4

Supplementary Figure 15

0.00	0.10	0.30	0.11	0.15	0.180	0.80	1.	0.00	0.08	0.09
0.02	0.12	0.56	0.14	0.17	0.170	0.65	1.	0.01	0.10	0.11
0.01	0.14	0.40	0.15	0.16	0.190	0.70	1.	0.02	0.12	0.14

Fig. S15a

0.	0.10	0.20	1.0	1.0	1.00	10.	15.0	17.0	38.	40.	50.
0.	0.01	0.05	0.2	0.3	0.12	3.	4.0	4.8	19.	21.	26.

Fig. S15b

0.3	0.50	0.250	1.0	1.0	1.00	1.6	2.5	2.9	5.0	6.0	8.0
0.0	0.20	0.125	0.3	0.5	0.48	0.5	0.8	1.0	1.8	2.0	3.0

Fig. S15c
